# Poxvirus infection triggers remodeling of host m⁶A epitranscriptome and benefits from the m⁶A regulatory responses

**DOI:** 10.1186/s12985-026-03160-y

**Published:** 2026-04-11

**Authors:** Taehyung Kwon, Demosthenes P. Morales, Abigale S. Mikolitis, Phillip M. Mach, Cheryl D. Gleasner, Sofiya N. Micheva-Viteva

**Affiliations:** 1 Biochemistry and Biotechnology Group (B-TEK), Bioscience Division, Los Alamos National Laboratory, Los Alamos, NM USA; 2 Center for Integrated Nanotechnologies (MPA-CINT), Los Alamos National Laboratory, Los Alamos, NM USA; 3 Genomics and Bioanalytics Group (B-GEN), Bioscience Division, Los Alamos National Laboratory, Los Alamos, NM USA

**Keywords:** Vaccinia virus, m⁶A epitranscriptome, Temporal transcriptomics, Post-transcriptional regulation, YTHDF1, Host-virus interaction

## Abstract

**Background:**

Understanding how host gene regulation responds to viral infection is essential for developing effective antiviral strategies. Emerging evidence suggests that host transcripts undergo dynamic chemical modifications to counteract viral invasion. Conversely, viruses that rely on nuclear transcription exploit host RNA methyltransferases to enhance mRNA export and translation. Orthopoxviruses, however, complete their entire replication cycle within compartmentalized cytoplasmic “factories” utilizing enzymes encoded by their large double-stranded viral DNA genomes. The dynamic interplay between host and poxviral epitranscriptome remains poorly characterized.

**Results:**

Using a temporally resolved model of Vaccinia virus (VV) infection, we investigated host-virus interactions through transcriptome and *N*6-methyladenosine (m⁶A) epitranscriptome whole genome sequencing. We found that host m⁶A modifications respond rapidly to VV infection, preceding the delayed transcriptional changes that emerge at later stages. Early m⁶A signatures included key innate immunity factors as well as host genes involved in transcriptional regulation, post-transcriptional modification, and protein ubiquitination. Functional assays validated two host factors with early m⁶A modification changes that are essential for VV infection: a m⁶A reader, YTHDF1, and a component of the SCF E3 ubiquitin ligase complex, FBXO31. The m⁶A gain on YTHDF1 enhanced its protein expression and promoted efficient VV replication. In addition, we identified previously unrecognized roles of FBXO31 and the SCF E3 ligase complex in supporting VV infection.

**Conclusion:**

Temporal profiling of the m⁶A epitranscriptome reveals how VV exploits host post-transcriptional regulatory pathways, specifically m⁶A RNA modification and protein ubiquitination. These findings highlight critical host factors co-opted during poxvirus infection and identify potential targets for therapeutic intervention.

**Supplementary Information:**

The online version contains supplementary material available at 10.1186/s12985-026-03160-y.

## Introduction

Infectious diseases caused by viral pathogens present a major global public health challenge, particularly when caused by emerging pathogens with pandemic potential [1–3]. The development of novel therapeutics to mitigate these threats requires a deep understanding of the complex host-virus interplay, rather than focusing solely on viral components, as this approach offers the potential for broad-spectrum antiviral strategies [4]. Regardless of their genome types, viruses hijack host transcription and translation machineries to replicate [5] and to evade host immune responses [6].

Moreover, research has demonstrated that viruses exploit host post-transcriptional machinery—particularly chemical modifications to RNA, such as *N*6-methyladenosine (m⁶A)—to modulate host responses and promote viral replication [7, 8]. Among RNA modifications, m⁶A stands out as the most abundant and extensively studied [9, 10]. The regulation of m⁶A modifications is mediated by specialized cellular proteins commonly referred to as “writers”, “erasers”, and “readers” [11]. These proteins add, remove, or interpret marks, thereby influencing RNA stability, splicing, localization, and translation [11, 12]. This regulatory process represents a key mechanism of host-virus interaction at post-transcriptional level.

The m⁶A modifications deposited on viral transcripts by host enzymes, such as the methyltransferase-like proteins METTL3 and METTL14, have been shown to enhance the nuclear export and translation of viral transcripts generated in the nucleus (e.g., retroviruses [13] and DNA viruses [14]). Similarly, m⁶A marks can alter the structure and binding affinity of viral RNAs, engaging various m⁶A writers, erasers, and readers, thereby modulating viral infectivity and host immune responses in cytoplasm-replicating viruses such as members of the *Flaviviridae* family [13, 15, 16]. Notably, emerging evidence indicates that host transcripts are also dynamically modified with m⁶A during viral infection, suggesting that viruses may reshape host m⁶A landscape to their advantage [13, 17–19].

Despite advances in understanding virus-induced post-transcriptional remodeling, far less is known about the m^6^A dynamics induced by cytoplasm-replicating DNA viruses, such as members of the *Poxviridae* family, which include several zoonotic *Orthopoxvirus* pathogens. Vaccinia virus (VV), the prototypic poxvirus and the vector used in the smallpox vaccine, serves as a well-established model for *Orthopoxvirus* replication and suppression of host immune defenses [20, 21]. VV replicates in the cytoplasm within endoplasmic reticulum-enclosed mini-nuclei [22]. Early suppression of host transcription and translation processes—a phenomenon commonly referred to as “host shutoff”—is a crucial part of the *Orthopoxvirus* pathogesis [21]. Although several VV proteins were implicated in this host shutoff stage by inhibiting RNA polymerase II, the precise mechanisms mediating this suppression, and the potential contribution of post-transcriptional modifications, remain unclear [21].

The m^6^A modification may serve as a sensitive indicator for the temporal progression of viral infection, occurring in parallel with changes in host gene expression. Furthermore, the interplay between m⁶A regulators (e.g., METTL, YTHDF proteins) and m^6^A-modified host transcripts during viral infection could reveal novel therapeutic vulnerabilities, offering promising avenues for host-directed antiviral strategies.

In this study, we investigated the temporal dynamics of both host and viral m⁶A epitranscriptomes, as well as global transcriptome. Using a time-course model of vaccinia virus (VV) infection in Vero cells—chosen for their broad susceptibility to diverse viral species [23]—we performed paired RNA sequencing (RNA-seq) and m⁶A-RNA immunoprecipitation sequencing (MeRIP-seq) [9] to capture changes in gene expression and m^6^A modifications at 2, 6, and 24 h post-infection (hpi). The MeRIP-seq method has been applied to identify transcriptome-wide m⁶A localizations that influence epigenetic regulation mechanisms in mammalian cells [9, 13–19]. However, some studies have questioned the reproducibility and specificity of MeRIP-seq for detecting peak locations and changes in methylation [24]. To address these limitations, alternative approaches—such as direct m⁶A sequencing using nanopore technology or multi-step chemical modification methods—have been proposed to pinpoint the exact positions of N⁶-methyladenosine on mRNA and lncRNA transcripts [25]. Since each m^6^A profiling technique has its strengths and disadvantages, the choice of methods for global epitranscriptome analysis is driven by the study’s research objectives.

The objectives of this study were to identify novel host factors regulating poxvirus replication by investigating the differentially expressed genes (DEGs), differentially m⁶A-modified regions (DMRs) on host viral transcripts, and their overlaps across distinct stages of VV infection. To achieve this, we developed an analytical workflow that integrates rigorous statistical analyses with stringent thresholds to define DMRs based on comparisons between MeRIP-enriched and input fractions. We further validated candidate regions through visual inspection of read-depth coverage plots across three or more biological replicates per condition. This approach helped overcome some of the limitations of the MeRIP-seq method [25] and improved the reproducibility and accuracy of DMR measurements in response to virus replication. We validated the MeRIP-seq findings using complementary methods, including direct RNA sequencing on the nanopore platform, single molecule RNA Fluorescence In Situ Hybridization (smRNA-FISH) technique, fluorescent immunocytochemistry, proteomics, and a novel proximity ligation assay that directly confirmed in situ m⁶A modifications and their impact on gene expression.

Our results reveal that host transcripts acquire m⁶A modifications immediately after viral entry, preceding detectable transcriptional changes. These early m⁶A signatures may serve as sensitive biomarkers of infection and point to host m⁶A and ubiquitination machinery as key drivers of poxvirus replication.

## Materials and methods

### Cell cultures and virus infection

Vero C1008 cell line (ATCC CRL-1586) was propagated in Eagle’s Minimum Essential Medium (EMEM; Thermo Fisher) supplemented with 10% fetal bovine serum (HyClone). Human dermal microvascular endothelial cells (HMEC-1, ATCC CRL-3243) were cultured in MCDB131 medium (Life Technologies) supplemented with 10% fetal bovine serum (HyClone), 10 ng/mL Epidermal Growth Factor (EGF; Thermo Fisher), 10 mM Glutamine (Thermo Fisher), and 10ng/mL Hydrocortisone (StemCell Technologies). LookOut Mycoplasma PCR detection kit (Millipore-Sigma) was used to verify that the cell cultures were mycoplasma-free. Vaccinia virus strain WR (ATCC VR-1354) was propagated in Vero C1008 cells inoculated at a multiplicity of infection (MOI) of 0.01. The virus was recovered from sonicated cell pellets collected when cytopathic morphology was observed in 50% of the infected cell cultures. Virus stocks were filtered through 0.45 μm syringe filters (Millipore-Sigma), and titers were determined using serial log dilutions, infections of Vero cells in 12-well culture plates, and fluorescent immunocytochemistry with an anti-VV antibody (Abcam, ab117453). SMER3, a selective inhibitor of DCF family E3 ubiquitin ligase, was obtained from Bio-Techne/TOCRIS Bioscience (Cat# 4375). Stock solutions, 10 mM in DMSO, were stored frozen (-20 C) for up to six months. Working solutions (1µM) were prepared immediately before VV infection experiments in cell culture media.

### Experimental design and m⁶A RNA immunoprecipitation protocol

**Fig. 1:**
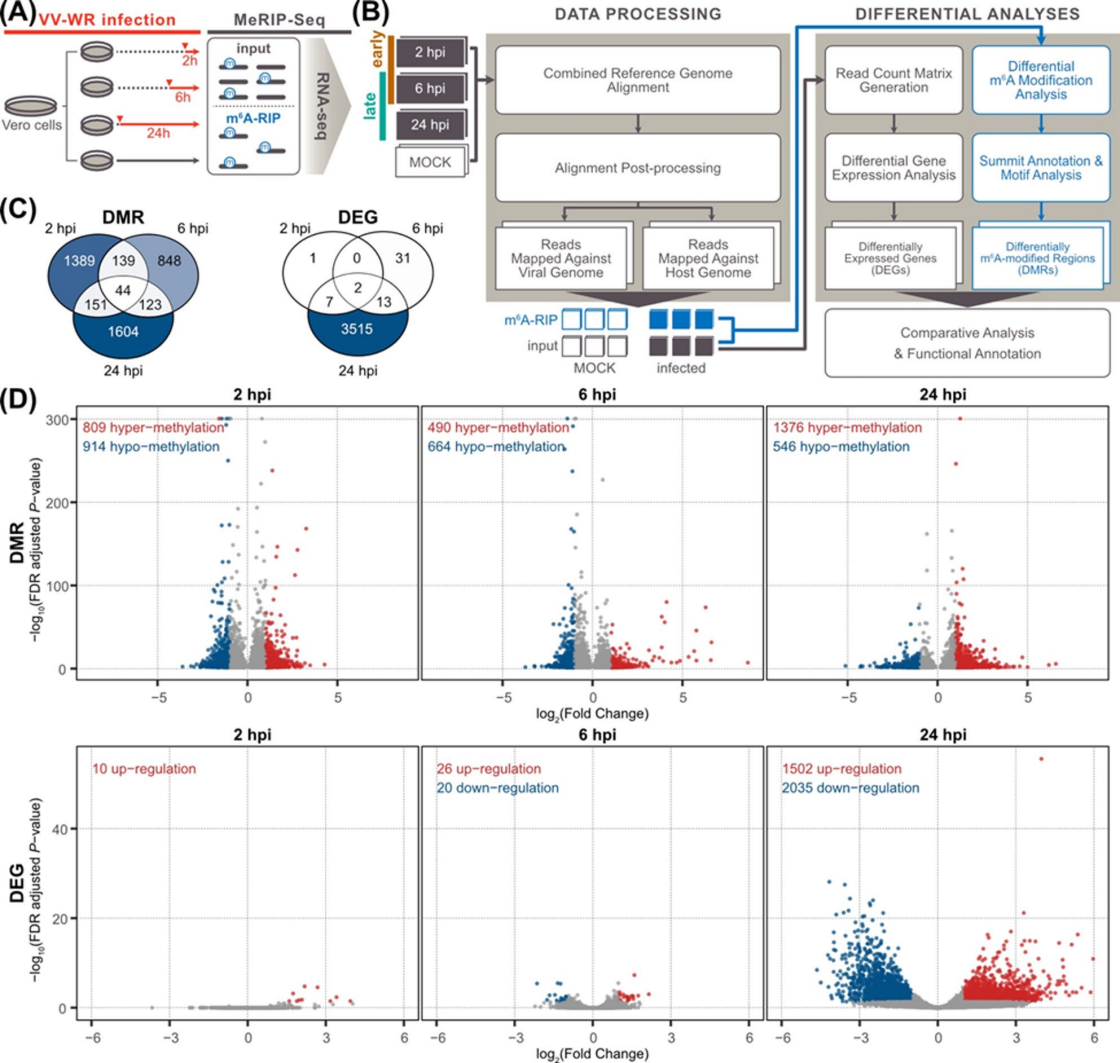
Summary of bioinformatics analyses on host RNA-seq and MeRIP-seq data. **(A)** A simplified diagram of the VV infection experiment. **(B)** A bioinformatic workflow for differential gene expression and differential m^6^A modification analyses. **(C)** Venn diagrams of differentially m^6^A-modified regions (left) and differentially expressed genes (right). **(D)** Scatter plots of differentially m^6^A-modified regions (top) and differentially expressed genes (bottom). Blue and red dots indicate statistically significant features: |log_2_(fold change)| ≥ 1 and FDR-adjusted *P*-value ≤ 0.05.

This experiment was designed to investigate the temporal dynamic of post-infection m⁶A RNA modifications. Vero cells (*Chlorocebus sabaeus* kidney) were chosen as the host of VV infection based on their permissiveness and extensive use for high virus titer production and vaccine development. For MeRIP-seq, we prepared four experiments from at least three independent infection experiment replicates: mock-infected experiment (MOCK, cells exposed to filtered supernatant of sonicated Vero cells) and three experiments representing 2 h post-infection (2 hpi), 6 hpi, and 24 hpi with VV-WR at MOI 1 (Fig. 1A). Total RNA was collected from each experimental sample via TRISOL-chloroform extraction and isopropanol precipitation of nucleic acids. DNA was removed by treatment with TURBO DNA-free Kit (Thermo Fisher). RNA was purified by precipitation with 80% ethyl alcohol in 150 mM Sodium acetate buffer (pH 5.2). RNA pellets were reconstituted in RNase free water (Thermo Fisher) and fragmented to 200–300 base pairs with 10mM ZnCl2, buffered with 10mM TrisHCl (pH 8).

To determine the relative m^6^A RNA methylation status we utilized *Fluorometric m*^*6*^*A RNA Methylation Assay Kit* (ab233491, Abcam). The assay is based on affinity capture and detection of m^6^A RNA. We followed the Manufacturer’s manual to quantitatively evaluate the percentage of m^6^A on total input RNA. The amount of input RNA per sample was in the range of 100–150 ng. The mass amount (ng) of m^6^A RNA was calculated relative to standard controls provided in the assay kit and the methylation status of each sample was calculated as a percentage of total RNA input.

RNA fragmentation was achieved by incubation at 70 °C for 5 min followed by rapid removal of Zn2 + by the addition of EDTA to final concentration 50mM and RNA precipitation with 80% ethyl alcohol supplemented with 400 µg/mL glycogen (Thermo Fisher, AM9510) in 150 mM Sodium acetate buffer (pH 5.2). Fragmented and purified RNA from each sample was divided to two fractions: input fraction with 0.1% of total RNA (input) and immunoprecipitated fraction with 99.9% of total RNA (m⁶A-RIP) (Fig. 1B). The m⁶A-RIP fraction was incubated with protein A/G magnetic beads coated with anti-m⁶A antibodies (EpigenTek, A-1801 and Cell Signaling Technology, D9D9W) for 4 h at 8 °C with agitation in immunoprecipitation (IP) buffer (150 mM NaCl, 10 mM TrisHCl (7.5 pH), 0.1% IGEPAL, and RNasin Plus RNase Inhibitor from Promega, N2611). Beads were washed twice (with 5 min incubation at 8 °C with agitation) in IP buffer, followed by two washes in low-salt IP buffer (50 mM NaCl, 10 mM TrisHCl (7.5 pH), 0.1% IGEPAL) and high-salt IP buffer (500 mM NaCl, 10 mM TrisHCl (7.5 pH), 0.1% IGEPAL). The m⁶A-RIP was eluted from the beads in 100µL RLT buffer from RNeasy MiniElute kit (QIAGEN) and column purified following the manufacturer’s protocol.

### MeRIP sequencing

The quality of input and m⁶A-RIP samples was determined with Agilent 2100 Bioanalyzer. SMARTer Stranded Total RNA-Seq Kit v2 - Pico Input Mammalian (Takara/Clontech, USA) was used for preparation of Illumina sequencing libraries following the manufacturer’s protocol without fragmentation with 16 cycles PCR amplification for the m⁶A-RIP RNA and 12 cycles for the input RNA. Size distribution of purified library was determined with Agilent 2100 Bioanalyzer and samples were sequenced on the Illumina NextSeq 500 platform with High Output Mode 75-cycle kit.

### Raw read filtering and alignment

For each sample, sequencing adapters and low-quality bases of the raw reads were trimmed from the raw reads using FastP v0.23.2 [26] with the following parameters: minimum base Phred quality of 20, minimum read length of 50, cut from the tail side, and a cut window size of 1. The filtered reads were then mapped to a combined FASTA of the *Chlorocebus sabaeus* reference genome (GCF_015252025.1) and the VV reference genome (GCF_000860085.1) using the STAR aligner v2.7.10b [27]. The *C*. *sabaeus* reference genome was indexed with exon awareness using its corresponding GTF annotation. Subsequently, we separated host-aligned reads and virus-aligned reads using Sambamba v1.0. For both host and viral alignments, we selected uniquely mapped reads using Sambamba v1.0 by removing PCR duplicates (sambamba-markdup) and filtering out reads with low mapping qualities (sambamba-view) [28]. For the downstream analyses, we generated read count matrices by counting mapped reads per gene using feature Counts from Subread v2.0.3 [29].

### Differential gene expression analysis

We investigated differential gene expressions between each VV-infected input samples (e.g., 2 hpi) and MOCK-treated samples. After filtering out lowly expressed transcripts (those with five or fewer mapped reads in more than three samples), we employed DESeq2 [30] to perform differential gene expression analysis. We selected significant DEGs based on |log_2_(fold change)| ≥ 1 and FDR-adjusted *P*-value ≤ 0.05.

### Differential m⁶A modification analysis

We investigated differential m⁶A modifications between each VV-infected samples (e.g., 2 hpi) and MOCK-treated samples using exomePeak2 v1.12.0 (https://github.com/ZW-xjtlu/exomePeak2). This program compares the m⁶A-RIP fraction and input fraction pairs of VV-infected samples (e.g., 2 hpi) with those of MOCK samples, while it also accounts for changes in gene expression. Based on the sequencing protocol, we applied strand-specificity (2nd _strand) and set the read fragment length of 150 bps in the exomePeak2 analysis. We selected significant DMRs based on |log_2_(fold change)| ≥ 1 and FDR-adjusted *P*-value ≤ 0.05.

### Functional annotation

To infer the m⁶A modification site for each DMR, we first generated read depth coverage graphs across nucleotide positions within each DMR using sambamba-depth [28]. We then inferred the m⁶A modification site by the position showing the highest read depth within the region. Using the inferred m^6^A modification site, we performed (i) peak annotation using annotatePeaks.pl and (ii) enrichment analysis for 5-mer sequence motifs using findMotifsGenome.pl of HOMER v4.11 package [31] with the following option: -rna, -mask, and -norevopp. We should note that a single transcript can contain multiple DMRs across its genomic loci. DMRs sharing the same locus (e.g., exon 1 of gene X) were deduplicated based on their FDR-adjusted *P*-values estimated by exomePeak2.

DMRs and DEGs were mapped together based on gene names. For host DMRs and DEGs, we performed functional annotation of GO terms and KEGG pathways using clusterProfiler v4.6.2 [32]. All statistical analyses and visualizations were performed in R, including but not limited to the Wilcoxon rank-sum test. Statistical significance was determined based on *P-*values, with corrections for multiple testing applied when necessary. Data visualization was performed using ggplot2 [33] package in R.

### Oxford nanopore direct RNA sequencing and analysis

Transcripts, isolated from MOCK-treated or VV-infected (MOI 1) Vero cells, were purified with NEBNext Poly(A) mRNA Magnetic isolation Module (E7490S, New England Biolabs, USA) following the Manufacturer’s protocol. Direct RNA sequencing kit (SQK-RNA004, Oxford Nanopore Tech, UK) was utilized to prepare libraries from 800 ng native mRNA following Manufacturer’s protocol. Each sample was sequenced on one MinION RNA Flow Cell (FLO-MIN004RA) using the Oxford Nanopore Tech (ONT) MinION Mk1C.

The ONT files in POD5 format consist of 5 million reads on average. We aligned ONT reads with minimap2 (“-x splice -k 14”) and basecalled m^6^A RNA bases using hac@v5.0.0 model on Dorado v0.8.1 [34]. We further sorted and indexed BAM file using Sambamba v1.0, then generated per-base m^6^A modification profiles using pileup function of Modkit v0.4.5 [35]. We selected the loci with three or more ONT reads aligned, which was further visualized using ggplot2 package in R.

### Real-time reverse transcription quantitative PCR assay

Total RNA was isolated from 10^6^ Vero or HMEC-1 cells with RNeasy Mini kit (QIAGEN, Germantown, MD) following the manufacturer’s protocol. DNA was removed with TURBO DNA-free kit (Thermo Fisher Scientific, USA). Input fraction (1 µg) was taken from total RNA prior to performing RNA IP with anti-N6-Methyladenosine monoclonal antibody (mAb) (D9D9W, Cell Signaling Technology, USA). The m⁶A-RIP fraction was prepared as described in *Experimental design and m⁶A RNA immunoprecipitation protocol* section of this study without the RNA fragmentation with ZnCl2 step. Input and m⁶A-RIP samples from three independent infection experiments were analyzed with Taqman RNA-to-CT 1-Step Kit (Applied Biosystems by Thermo Fisher Scientific, USA) in a 25 µl volume according to the manufacturer’s protocol. The reactions were run on QuantStudio-3 Real-Time PCR System (Applied Biosystems by Thermo Fisher Scientific, USA) in triplicates. TaqMan probes for detection of *YTHDF1* (target gene) and GAPDH transcripts (sample reference control) in Vero cells included Rh02873387_m1 and Rh02621745_g1, respectively. *YTHDF1*, *AKT1*, *FBXO31*, *METTL3*, and *ACTb* transcript levels in human (HMEC-1) cells were analyzed with Hs00697331_m1, Hs00982883_m1, Hs00375554_m1, Hs00219820_m1, and Hs01060665_g1 Taqman probes, respectively (Applied Biosystems by Thermo Fisher Scientific, USA). Double delta Ct analysis was used to calculate fold change of real-time differential gene expression in VV-infected versus MOCK-treated cells (2^−ΔΔCt^) in both input and m⁶A-RIP fractions. Real-time differentially m⁶A modification values were obtained as ratio between transcript levels in the m⁶A-RIP versus input fractions in VV-infected cells normalized to the corresponding ratio in the MOCK-treated samples.

### Gene knockdown using RNA interference

*FBXO31* transcripts in Vero and human skin endothelial cells (HMEC-1) were depleted with Silencer^®^Select synthetic RNA interference duplexes (siRNA) ID n295386 (Invitrogen for ThermoFisher scientific, USA) targeting transcript variants 1 and 2 at exons 10 (4799 nt mRNA location) and 9 (5051 nt mRNA location), respectively. The knock down of *YTHDF1* and *METTL3* transcripts was performed with Silencer^®^Select siRNA reagents s29743 and s32143, respectively. Non-targeting control siRNA reagent (4390843, Invitrogen for ThermoFisher scientific, USA) was used for treatment of reference control samples. In all experiments, cell cultures were transfected with 20nM siRNA in Lipofectamine RNAiMAX Transfection Reagent (Invitrogen for ThermoFisher scientific, USA), following the manufacturer’s protocol, and were incubated for 18 h prior to VV infections.

### Single molecule RNA fluorescence in situ hybridization assay

Fluorescent probes, specific to *YTHDF1* transcript, were purchased from LGC BioSearch Technologies (sequences of 48, Quasar 570 labeled probe sets are listed in Table S6). Stellaris RNA-FISH protocols were applied for Sequential immunofluorescence with anti-N6-Methyladenosine Rabbit mAb (D9D9W), secondary anti-rabbit AlexaFluor 488 Ab (A11034, ThermoFisher Scientific, USA), and Quasar 570 labeled probes to YTHDF1 mRNA. Briefly, at indicated time points of treatment, cells were fixed in 4% paraformaldehyde for 15 min and incubated in 3% BSA blocking buffer (BSA blocking buffer, ThermoFisher Scientific, USA) with 0.2% Triton for 1 h at 37 °C. Fixed and permeabilized cells were incubated with D9D9W anti-m⁶A antibody (diluted 1:200) in 1% BSA in PBS overnight at 4 °C, followed by four washes in PBS (with 0.05% Tween) and 1 h incubation at 37 °C with secondary, anti-rabbit IgG-AlexaFlour488. Secondary antibody was removed with four washes in PBS-Tween buffer and cells were incubated second time in 4% PFA for 10 min. Prior to performing RNA hybridization, cells were incubated for 1 h at 37 °C in Wash buffer A (LGC BioSearch Technologies, USA) supplemented with 10% formamide. The pre-hybridization buffer was replaced with hybridization buffer (LGC BioSearch Technologies, USA) supplemented with 10% formamide, 1µM Quasar 570 labeled probe set, and 1U of RNasin Plus RNase Inhibitor (Promega, N2611). The hybridization reaction was carried out overnight at 37 °C in a humidified chamber. Hybridization buffer was replaced with Wash Buffer A and incubated under foil for 30 min at 37 °C. The buffer was replaced with Wash Buffer A containing 1 ug/mL DAPI (ThermoFisher) and incubated for 30 min at 37 °C under foil. DAPI was removed and replaced with Wash Buffer B and incubated for 5 min at room temperature. Wash Buffer B was replaced with a 2X solution of SSC buffer (ThermoFisher Scientific, USA) and samples were immediately imaged to avoid loss of signal due to RNA degradation.

### Microscope imaging, cell segmentation, and counting

Microscope imaging was performed on an Olympus IX83 with a UPLAPO100XOHR 100 × 1.50 NA oil objective lens and a Hamamatsu ORCA-Flash4.0 detector. Mirror cubes were used for DAPI, Cy3 and Cy5 with emission wavelengths of 455, 580 and 670 nm, respectively. 10 images per well were collected with multiple cells in the field of view. Z-stack images were collected at 0.46 μm per step spanning 15 μm in the Z-axis. Image voxel sizes were 0.26 ⋅ 0.26 ⋅ 0.46 μm for x, y and z, respectively. Image channels were split and exported as 16-bit grayscale TIFF files using ImageJ [36].

Cellpose v3.0.6 [37] was used to generate labeled, segmented cell images of 2D maximum intensity projected images of anti-*N6*-Methyladenosine Rabbit mAb (D9D9W, Cell Signaling Technology, USA) and secondary anti-rabbit AlexaFluor 488 Ab (A11034, ThermoFisher Scientific, USA) that defined cellular boundaries. The “cyto3” pretrained model was selected with a flow threshold of 0.4 and pixel diameter of 200. ImageJ was used to perform a standard deviation projection on the smRNA-FISH channel images to increase signal contrast and remove non-specific signals. An in-house python script pipeline was used to analyze the smRNA-FISH datasets. The Big-FISH v0.6.2 python package [38] was used to extract the coordinates of spots on the smRNA-FISH images. Images were read using the scikit-image v0.20.0 package [39]. A threshold was determined for the sets of images based on the projected smRNA-FISH images such that signals below the spot signatures were removed, additionally a spot radius of 520 nm and an alpha value of 0.7 was used as input parameters.

### Proximity ligation assay for detection of m^6^A peaks on RNA transcripts

The assay was adapted from a previous application designed for the detection of RNA-protein interactions [40]. RNA probes (Integrated DNA Technologies, IDT) consisted of 40 deoxynucleotide (nt) sequences complementary to the *YTHDF* transcripts of the *C. sabaeus* genome, followed by a 20-deoxyadenosine (dA) linker and a 25-nt primer sequence for rolling circle amplification. The rolling circle primer (25 nt) was antisense to the plus PLA probe conjugated to the anti-rabbit antibody (Duolink In situ PLA Probe, DUO92002, Sigma-Millipore): TACTGTCTTGATCTGTGAGAA. A total of **10** RNA probes were used, with target-specific sequences designed using the RNA-FISH algorithm from LGC BioSearch Technologies. Sequences of oligonucleotide probes used in this study are listed in Table S6.

Cells were fixed and permeabilized, then blocked with 3% BSA in phosphate-buffered saline (PBS) for 1 h at room temperature, followed by incubation with an anti-m⁶A antibody (Cell Signaling Technology, D9D9W) overnight at 4 °C. The antibody was washed off with PBS containing 0.01% Tween-20 (PBST) four times, and cells were fixed with 4% paraformaldehyde for 10 min. Cells were then incubated in Wash Buffer A (LGC BioSearch Technologies, USA) supplemented with 10% formamide. Hybridization of 1 µM PLA probes was performed as described in the smRNA-FISH hybridization section of this study. After probe removal, cells were rinsed twice with Wash Buffer A and incubated in 40 µL of Duolink Blocking Solution. All subsequent PLA steps were performed according to the manufacturer’s instructions using the Duolink In situ Detection Reagents Orange (DUO92007, Sigma-Millipore) and the Duolink In situ PLA Probe Anti-Rabbit PLUS (DUO92002, Sigma-Millipore). Finally, nuclei were counterstained with DAPI, and samples were mounted with FluorSave reagent (Sigma-Millipore) under glass coverslips. Images were acquired using an automated ZEISS Axio Observer.Z1 microscope with a 65× objective.

### Western blot and fluorescence immunocytochemical assays

Western Blot (WB) analysis was performed on 10 µg total protein isolated from cell cultures solubilized in RIPA Lysis and Extraction Buffer (ThermoFisher Scientific [TFS], Grand Island, NY). Protein samples were resolved by Tris-Glycine-SDS PAGE using 4–15% gradient Mini-PROTEAN gels (BioRad, Hercules, CA). Proteins were transferred from the PROTEAN gels onto PVDF membranes (Millipore, Billerica, MA). Prior to overnight incubation (at 4⸰C) with primary antibodies, PVDF membranes were blocked for 1 h with 5% non-fat milk in TBST (25 mM Tris [pH7.6], 137 mM NaCl, and 0.2% Tween20). The following primary antibodies were used in this study: anti-YTHDF1 (P5HB-23, Invitrogen-TFS), anti-METTL3 (E3F2A, CST-86132, Cell Signaling Technology, Danvers, MA), anti-Actin (ACTN05(C4), Invitrogen-TFS), and anti-Vaccinia virus VP39 (ab87387, abcam-Danaher, Waltham, MA). Post incubation (1 h at room temperature) with the corresponding HRP-conjugated secondary antibodies, immunoreactive proteins were visualized with Luminol reagent (Santa Cruz Biotechnology) on BioRad Molecular Imager. Quantitative analysis of protein band intensities was performed with Image Lab software.

Immunocytochemical analysis was performed on fixed (4% paraformaldehyde) and permeabilized (0.2% Triton in PBS) cell cultures that were incubated overnight with primary anti-FBXO31 (NBP1-19088, Novus Bio, Centennial, CO), anti-YTHDF1 (P5HB-23, Invitrogen-TFS), and anti-Vaccinia virus (PA1-7258, Invitrogen-TFS). Alexa Fluor488 or Alexa Fluor555 goat anti-rabbit IgGs were used to fluorescently label VV and cellular proteins. Images were obtained with Zeiss Axio Observer.Z1 using ZenPro software.

### Proteomics data acquisition and analysis

All proteomics data were collected using a Dionex 3000 UHPLC (Thermo Fisher Scientific) coupled to a high-resolution tribrid Eclipse mass spectrometer (Thermo Fisher Scientific). Samples were normalized by protein content within the workflow, allowing for equal volume injections of four µL to be made. Chromatography was performed using 0.1% formic acid in water (A) or acetonitrile (ACN) (B) on a reversed phase EasySpray C18 column (20 μm x 750 mm, Thermo Fisher Scientific) heated to 55° C with a flow rate of 0.25 µL/min and an acquisition time of 65 min. The gradient began at 5% B until 3 min where it increased to 10% B, followed by an increase to 35% B by 50 min, 70% by 52 min, and 90% B by 53 where the column was washed for three min and re-equilibrated to 5% for 3 min. Data collection was performed in positive mode via data-dependent acquisition (DDA), using a two s cycle time with an N of 20 and an exclusion list of 60 s. Data were acquired with a scan range from *m*/*z* 375-1,500, a resolution of 240,000, and MS/MS was acquired using HCD followed by ion trap detection with a scan rate set to turbo.

Proteomics data analysis was performed by first downloading the *Homo sapiens* reference database (UP000005640, downloaded on August 20th, 2024) from UniProt (conical and isoforms) [41]. Processing and protein assignments were performed with Proteome Discoverer v2.5 [42] using a label-free quantification workflow with Sequest HQ and Percolator using default settings where FDR-adjusted *P*-value < 0.05 was considered as medium confidence and FDR-adjusted *P*-value < 0.01 as high confidence.

PEAKS Studio 11 was used for comparison and de novo using the PEAKS DB (In-depth de novo assisted search) workflow [43] with the *Homo sapiens* reference database (UP000005640). Replicates were combined under a single sample and a precursor mass error tolerance of 15 ppm was allowed. The digestion mode was set to semi-specific with allowable missed cleavages of up to 3. PTM Search and Spider were enabled with default settings. The reporter filter tolerances used default settings for a peptide spectrum match FDR of 1.0%, a protein − 10log(*P*-value) of ≥ 15.0, and an average local confidence percentage ≥ 50.0%.

## Results

### Changes in host m⁶A epitranscriptome precede genomic responses

To characterize the temporal dynamics of host gene regulation at 2, 6, and 24 h post-infection (hpi) with vaccinia virus (VV), we profiled transcriptome-wide m⁶A RNA modifications and gene expression in Vero cells (*Chlorocebus sabaeus*) (Fig. 1A). We employed paired sequencing of m⁶A RNA immunoprecipitation fraction (hereafter referred to as **m⁶A-RIP**) and the corresponding RNA-seq input fraction (referred to as **input**). These time points were selected to represent distinct stages of VV infection: 2 hpi (early infection, pre-replication), 6 hpi (mid-infection, viral genome replication and virion formation), and 24 hpi (late infection, virion release). For each time point, we identified differentially expressed genes (DEGs) and differentially m⁶A-modified regions (DMRs) (Fig. 1B).

The differential m⁶A modification analysis revealed 4,298 DMRs on 1,938 loci within 1,656 genes (Table S1) across all three time points of VV infection (Fig. 1C), the majority of which detected in exonic regions (Figure S1). The *GACGA* sequence motif was the most significantly enriched across DMRs (Figure S2 and Text S1). In contrast, differential gene expressions in host transcripts were mostly detected at 24 hpi (Fig. 1C). Specifically, 3,515 of the 3,569 DEGs were detected exclusively at 24 hpi, while 1,723 and 1,154 DMRs were detected at 2 hpi and 6 hpi, respectively (Fig. 1C). We observed a decrease in m⁶A modification level at 6 hpi compared to 2 hpi and 24 hpi, consistent across the number of DMRs (Fig. 1C), in situ fluorescent immunocytochemistry (Figure S3A), and in vitro quantitative m⁶A RNA methylation assay (Figure S3B). Together, these results suggest the early dynamic remodeling of host m^6^A landscape during VV infection, with a transient reduction in m⁶A modifications at 6 hpi, followed by host gene expression changes in the late stage of VV infection.

For downstream analysis, we grouped DMRs and DEGs based on the directions of their fold changes. DMRs were defined as hyper-methylated (i.e., gain of m⁶A modifications) and hypo-methylated (i.e., loss of m⁶A modifications), while DEGs were classified as up-regulated and down-regulated (Fig. 1D).We observed a weak correlation between the directions of differential m^6^A modifications and gene expressions (Pearson’s *r* = 0.28; *P*-value = 1.56E-09) in host transcripts that exhibited changes in both m^6^A modification and gene expression (hereafter referred to as DMEGs) (Figure S4). Notably, most of the DMRs detected at 2 hpi and 6 hpi (99.5%) did not show simultaneous changes in their gene expression, and the majority of DEGs at 24 hpi (89.12%) were not differentially m⁶A-modified (Figure S4). Thus, VV infection induces temporally distinct regulations between host transcriptional and post-transcriptional layers, represented by the dynamic and consistent changes in host m^6^A profile and the late changes in host gene expression.

### Host m⁶A signatures reveal early determinants of host-virus interactions

MeRIP-seq analysis revealed that 89.4% of the DMRs were uniquely detected at a single time point (Fig. 1C), highlighting the highly dynamic and time-sensitive nature of host m^6^A modifications. To identify persistent m^6^A signatures, we focused on DMRs shared between adjacent time points (Fig. 1B). This analysis revealed 139 early-phase DMRs (shared between 2 and 6 hpi), 123 late-phase DMRs (shared between 6 and 24 hpi), and 44 persistent DMRs (shared across all three time points). Notably, many of these consistent m^6^A modification changes are associated with previously established host-virus interaction (Text S2).

**Fig. 2:**
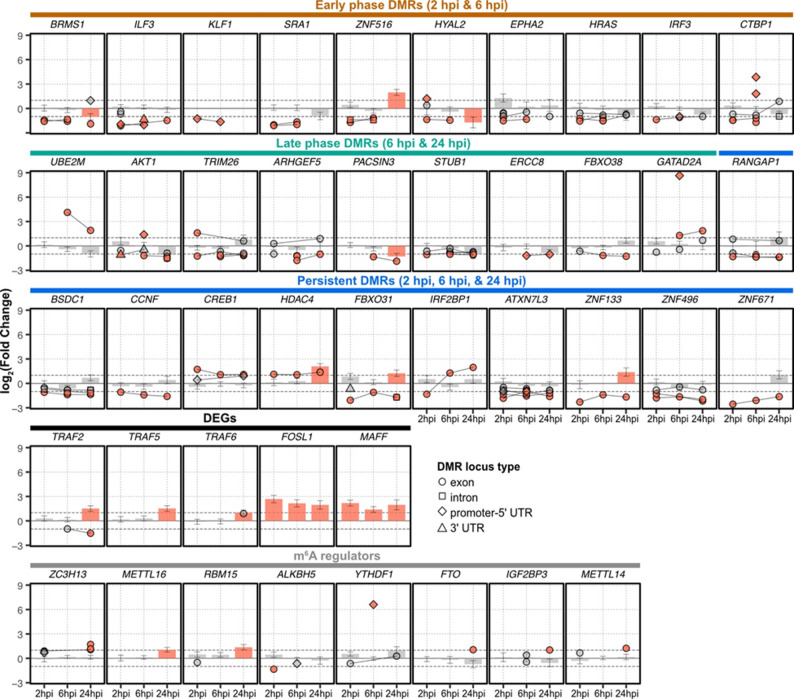
Key host genes showing changes m^6^A modifications or gene expressions. Each plot depicts log_2_(fold change) of differential m^6^A modifications (dots) and differential gene expressions (bars) across three time points of infection. Orange-colored features indicate statistical significance. Dots connected with lines indicate differential m^6^A modifications detected in the same locus

Among the **early-phase DMRs**, we observed that several host factors previously identified as enhancers of viral entry or regulators of innate immunity—including *HYAL2* [44], *HRAS* [45], *EPHA2* [46, 47], *ILF3* [48, 49], *IRF3* [50], and *CTBP1* [51, 52]—showed loss of m^6^A modifications in their coding regions without corresponding changes in gene expression levels (see *Early phase DMRs* in Fig. 2, denoted by orange circles; Text S2). Among the **late-phase DMRs**, transcripts encoding cellular factors involved in antiviral immunity (*UBE2M* [53]) and viral replication (*AKT1* [54, 55]; *TRIM26* [56, 57]) exhibited consistent m^6^A modification changes (see *Late phase DMRs* in Fig. 2; Text S2). Similarly, **among 44 persistent DMRs** were host factors previously reported to enhance viral replication (*RANGAP1* [58], *BSDC1* [59], and *CREB1* [60–62]) or suppress viral infectivity (*CCNF* [63]) (see *Persistent DMRs* in Fig. 2 and Text S2). Notably, *HDAC4*, a regulator of the type I interferon (IFN) pathway and a known VV restriction factor, displayed a two-fold increase in m^6^A peaks enrichment across all time points followed by transcriptional up-regulation at 24 hpi [64, 65] (Fig. 2).

In short, VV infection rapidly alters post-transcriptional modifications on host transcripts involved in host-virus interaction, underscoring the importance of the host m⁶A epitranscriptome profiling in detecting early signatures of VV infection.

### Host transcriptional responses peak at the late stages of viral replication

**Fig. 3:**
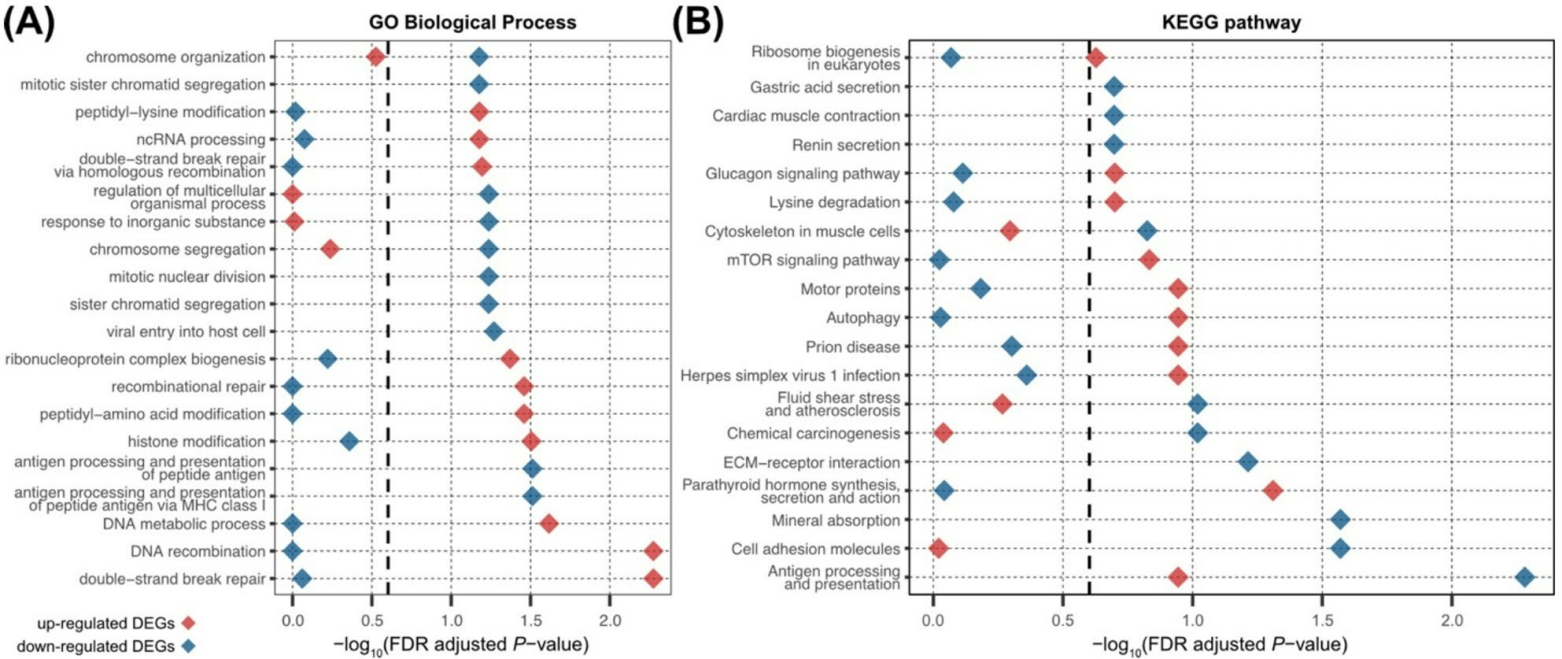
Gene set enrichment analysis result of host differentially expressed genes detected at 24 hpi. Ten most statistically significant **(A)** GO Biological Process terms and **(B)** KEGG pathways were selected for up-regulated genes (red) or down-regulated genes (blue). Dashed lines display FDR-adjusted *P*-value cutoff of 0.25

We performed Gene Set Enrichment Analysis (GSEA) of Gene Ontology (GO) terms for DEGs detected at 24 hpi and found significant functional enrichment in host genes related to poxvirus replication. Consistent with their role in the DNA virus replication strategies and host immune modulation, GO terms associated with double-strand break repair and post-translational modification were represented by up-regulated DEGs [66, 67]. Down-regulated DEGs included genes involved in antigen processing and presentation (FDR-adjusted *P*-value ≤ 0.25; Fig. 3A and Table S2). GSEA of Kyoto Encyclopedia of Genes and Genomes (KEGG) pathways further demonstrated enrichment of DEGs in antigen processing and presentation pathways, observed among both down-regulated (FDR-adjusted *P*-value = 5.20E-03) and up-regulated genes (FDR-adjusted *P*-value = 0.11) (Fig. 3B and Table S3). In addition, genes associated with dsDNA virus replication (Fig. 3B, herpes simplex virus type 1, FDR-adjusted *P*-value = 0.11), and ubiquitin E3 ligase-encoding genes *TRAF2*, *TRAF5*, and *TRAF6* (Fig. 2) were up-regulated at 24 hpi. These genes are known to modulate IFN signaling and viral entry [68–70].

Furthermore, two transcription factors (FOSL1 and MAFF) showed persistent up-regulation of gene expression across all three time points (Fig. 2). Both transcription factors belong to the activator protein-1 (AP-1) family, supporting either innate immune responses or viral replication in single-stranded RNA and double-stranded DNA viruses [71–74]. This result confirms that VV replication is dependent on host transcriptional machinery. Together with the m^6^A survey, host gene expression profile provides complementary insights into the VV-host mechanistic interactions.

### VV infection impacts host m^6^A regulatory responses

Motivated by prior reports highlighting the critical role of m⁶A modification machinery in viral replication [75], we next examined 19 genes regulating m⁶A modification machinery. Two m⁶A writer genes, *METTL16* and *RBM15*, were up-regulated at 24 hpi (see *m⁶A regulator* of Fig. 2), coinciding with the overall predominance of hyper-methylated DMRs at this time of VV infection (Fig. 1D). Additionally, we detected differential m⁶A modifications in several transcripts, including two erasers (*ALKBH5* and *FTO*), two additional writers (*METTL14* and *ZC3H13*), and two readers (*YTHDF1* and *IGF2BP3*) (see *m⁶A regulator* of Fig. 2).

**Fig. 4:**
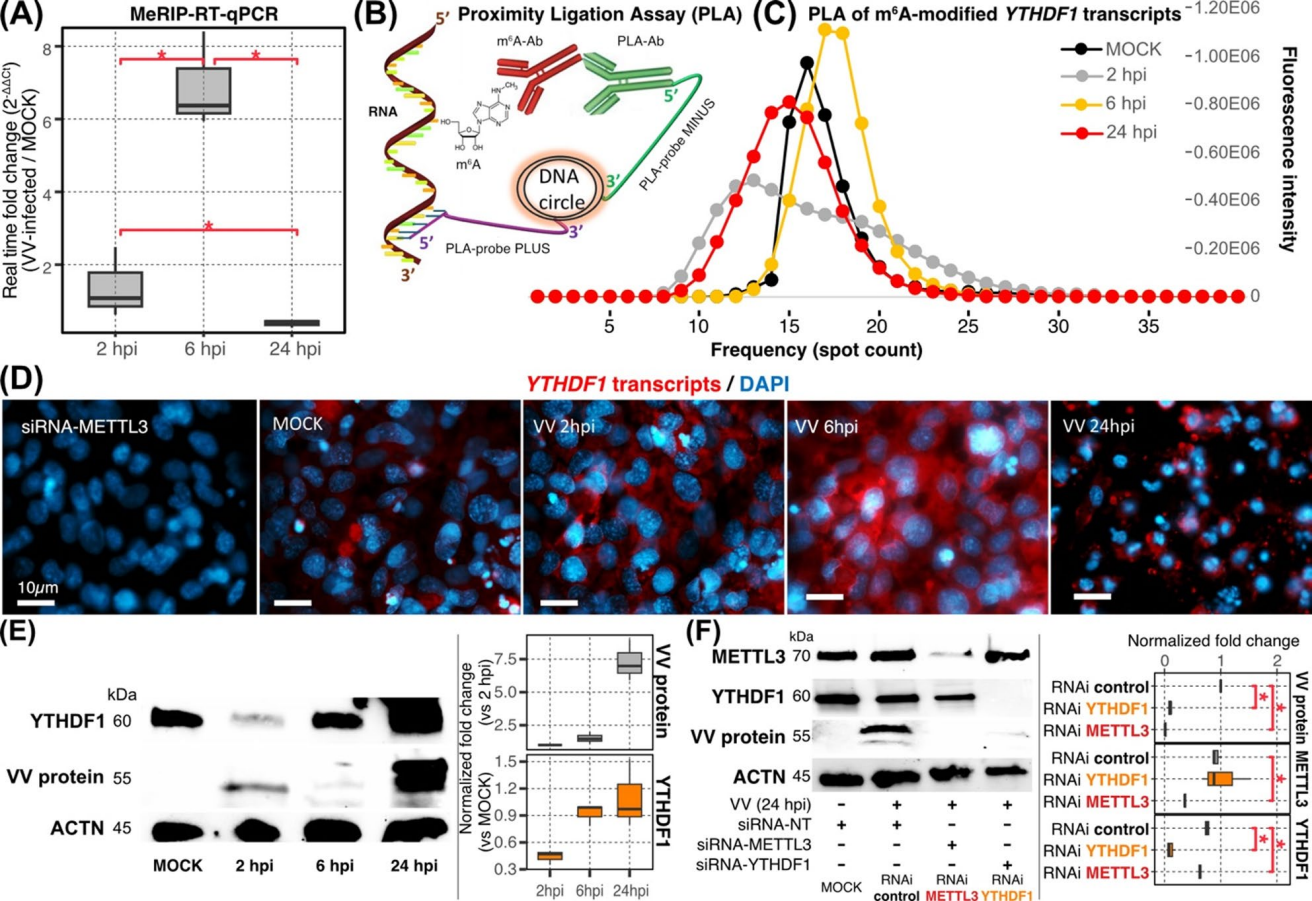
Interplay between VV replication and m^6^A regulators. **(A)** MeRIP-RT-qPCR analysis of *YTHDF1* transcript levels in VV-infected Vero cells. *YTHDF1* and *Actin-beta* transcript abundances were calculated in anti-m^6^A-antibody enriched RNA samples with real-time quantitative PCR. Fold change in transcript levels was calculated as ratio between VV-infected to MOCK-treated samples. Shown are the average and standard deviations calculated from four independent infection experiments. Red asterisks indicate statistical significance (Wilcoxon rank-sum test *P*-value ≤ 0.05). **(B)** A diagram of proximity ligation assay (PLA) designed to fluorescently label m^6^A modifications on *YTHDF1* mRNA. Oligonucleotide probes, specific to *YTHDF1* mRNA, have 25-nt, 5’-sequence that is complimentary to the 3’-nucleotide tail of PLA secondary, anti-rabbit antibody. Affinity binding of secondary PLA antibody to primary anti-m^6^A antibody brings the *YTHDF1* mRNA hybridization probes close to the m^6^A modifications thus creating conditions for rolling-circle DNA amplification and accumulation of fluorescent signal [40]. **(C)** Quantitative analysis of florescence intensity and distribution within cell populations infected with VV is performed on PLA images utilizing ZEISS ZenPro2.0 software. **(D)** Fluorescence imaging of PLA-labeled m^6^A- *YTHDF1* mRNA in MOCK-treated and VV-infected Vero cells. PLA fluorescence imaging was performed in Vero cells treated with 20 nM siRNA targeting *METTL3* transcripts. This is a control for the affinity reagent and PLA fluorescence signal specificity. Shown are images from a representative VV infection experiment. The m^6^A-modified *YTHDF1*transcripts are shown in red and cell nuclei, labeled with DAPI, are depicted in blue. The white scale bar corresponds to 10 μm. **(E)** Western Blot (WB) analyses of YTHDF1 and VV proteins in Vero cells across three post-infection time points. In each sample, YTHDF1 protein levels were normalized to Actin-beta and calculated as ratio relative to MOCK-treated controls. **(F)** WB analyses of YTHDF1, METTL3, and VV proteins in HMEC-1 cultures at 24 hpi of VV infection. In *YTHDF1* or *METTL3* knockdown samples, siRNA reagents (20 nM) were added 20 h prior to VV infection. Fold changes of protein levels were calculated as the ratios of the corresponding treatment to MOCK-treated siRNA-NT. Bar graphs show averages and standard deviations from normalized protein levels calculated from three independent experiments. Statistical significance (Wilcoxon rank-sum test *P*-value ≤ 0.05) is indicated with red asterisks.

Specifically, we observed a transient and significant gain of m⁶A modifications in the 5′ untranslated region (UTR) of *YTHDF1* transcripts at 6 hpi (log_2_(fold change) = 6.61; Figs. 2 and S5A). YTHDF1 is a m⁶A-RNA binding protein known to promote translation [76]. Furthermore, the base-resolution m^6^A survey with Oxford Nanopore direct RNA sequencing supported de novo accumulation of m^6^As on *YTHDF1* 5’ UTR at 6 hpi (Figure S5B). This steep increase in m^6^A modifications was additionally confirmed by methylated RNA immunoprecipitation followed by reverse transcription quantitative PCR (MeRIP-RT-qPCR). Compared to MOCK-treated Vero cells, higher levels of m^6^A-modified *YTHDF1* transcripts were detected at 6 hpi (fold change = 6.91 ± 1.32 L), followed by a decline at 24 hpi (fold change = 0.38 ± 0.11) (Fig. 4A).

We further introduced a novel assay to quantify m⁶A-modified *YTHDF1* transcripts in situ. We adapted proximity ligation assay (PLA) followed by fluorescence imaging analysis, leveraging *YTHDF1* specific oligonucleotide probes that bridge RNA template with anti-m⁶A antibody interactions to generate fluorescence signal illuminating the m⁶A-modified *YTHDF1* transcripts (Fig. 4B). The PLA fluorescence intensity, corresponding to m⁶A modifications on the *YTHDF1* transcripts, was higher at 6 hpi than 2 hpi or 24 hpi (Fig. 4C). Simultaneously, the PLA frequency (fluorescent cell count) increased at 2 hpi with low PLA intensity, indicating increased number of cells with m⁶A modifications on the *YTHDF1* transcripts in the course or VV replication and higher concentration of the m⁶A modifications observed at 6 hpi (Fig. 4C). The observed decrease of fluorescence intensities at 24 hpi compared to 6 hpi may be caused by the reduction of cytoplasmic contents in the peak of viral particle formation (Fig. 4C and D). Utilizing RNA interference (RNAi) we suppressed *METTL3* transcripts (encoding for Methyltransferase-like 3 m^6^A-writer) and observed no fluorescence generated by the PLA, thus validating the specificity of antibody binding to m⁶A modification sites (see *siRNA-METTL3* in Fig. 4D). In contrast, total *YTHDF1* transcript levels determined by RNA-seq (Fig. 2) and RT-qPCR (Figure S6) were up-regulated at 2 hpi (fold change = 2.73 ± 2.35) and 24 hpi (fold change = 6.65 ± 1.27) and down-regulated at 6 hpi (fold change = 0.38 ± 0.17). We further validated these results with single molecule RNA fluorescence in situ hybridization (smRNA-FISH) assay. The average count of *YTHDF1* transcripts per cell, approximated from three independent infection experiments, confirmed gene activation at early (2 hpi) and late (24 hpi) stages of VV infection, while at the 6hpi there was a relative decrease in the smRNA-FISH spot counts (Figure S7). Altogether, via alternative experimental protocols we detected inverse correlation between the accumulation of m⁶A- modifications and *YTHDF1* transcript levels at 6 hpi.

### Gain of m^6^A modification on *YTHDF1* transcript promotes its translation

Next, we examined the impact of m^6^A modification dynamics on YTHDF1 protein expression in VV-infected cells. Time course analysis with fluorescence immunocytochemistry confirmed the VV spread throughout the Vero cell populations (Figure S8). Western blot analysis on total protein, isolated from matching sets of VV infected Vero cells, confirmed the accumulation of viral protein detected at 2, 6, and 24 hpi. Quantitative analysis of protein levels via WB densitometry showed a distinct decrease of YTHDF1 shortly upon infection (2 hpi) followed by a consistent accumulation at 6 and 24 hpi (Fig. 4E).

Altogether, our results indicate that YTHDF1 protein levels become depleted immediately upon VV infection and the observed increase of m^6^A modifications in the 5’UTR *YTHDF1* at 6 hpi coincides with stimulation of translation. This may be a compensatory mechanism for protein loss early upon infection. Collectively, these results support a role for m⁶A modifications within the 5′ UTR of *YTHDF1* transcripts in driving the translation process [77].

### Host m^6^A machinery facilitates VV replication

Previous studies have suggested that components of the host m⁶A machinery are essential for viral protein expression [78, 79]. To directly evaluate their role in the VV life cycle, we depleted key m^6^A regulators—a m^6^A-reader (**YTHDF1*)*** and a m^6^A-writer (**METTL3**), known to govern the *N*6-methyladenosine modification processes across host transcriptome—in VV-infected cells using synthetic, RNA interference reagents (RNAi). Due to limited availability of RNAi reagents that are efficient in transcript knockdown within the *C. sabaeus* host genome, we introduced the human skin endothelial cell line, HMEC-1, as an alternative model of VV infection. Simultaneously, HMEC-1 validates the role of m^6^A machinery in VV replication across different host species and tissue types while providing the innate immunity that is lacking in Vero cells (e.g., type I IFN defenses).

Non-targeted, global proteomics analysis of VV-infected HMEC-1 cells revealed YTHDF1 protein dynamics comparable to the trajectory of YTHDF1 protein expression in VV-infected Vero cells (Fig. 4E): depletion of YTHDF1 early upon infection of HMEC-1 cultures (6 hpi) was followed by a compensatory increase in protein abundance at 24 hpi (Figure S9 and Table S4).

Next, we utilized RNAi for a transient knockdown of *YTHDF1* and *METTL3* transcripts and analyzed VV protein accumulation in HMEC-1 cultures via WB (Fig. 4F). Compared to control samples treated with non-targeting siRNA, both siRNA-YTHDF1 and siRNA-METTL3 treated samples had significantly reduced VV protein levels: fold changes of 9.11E-02 (Wilcoxon rank-sum test *P*-value = 3.18E-02) in siRNA-YTHDF1 and 2.03E-02 (Wilcoxon rank-sum test *P*-value = 2.97E-02) in siRNA-METTL3 (Fig. 4F). The YTHDF1 or METTL3 protein levels were significantly reduced in the RNAi-treated samples, thus demonstrating the dependence of VV replication on host m^6^A modification machinery. We further validated the WB results with florescence imaging of VV infected HMEC1 cultures subjected to transient *YTHDF1* knockdown and observed a dramatic decrease of viral infection within cell cultures with RNAi-reduced expression of the m^6^A reader (Figure S10). Collectively, the results presented here suggest that the host m^6^A regulators are required for VV protein production and completion of infectious viral cycle.

**Fig. 5:**
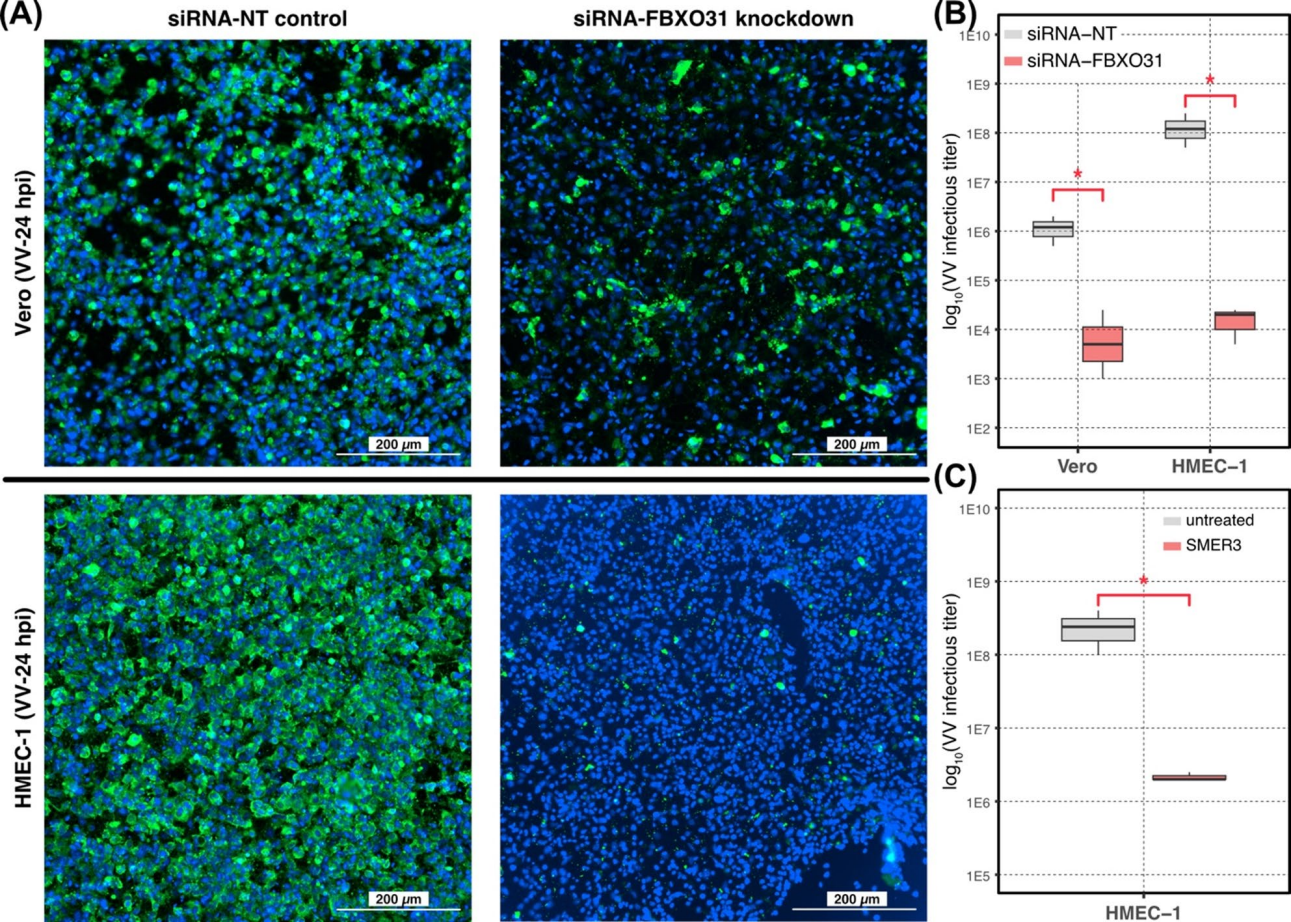
Results of various inhibition quantitative assays for *FBXO31* and SCF E3 ubiquitin ligase. **(A)** Fluorescence immunocytochemistry image analysis with antibodies targeting VV proteins in Vero (top) and HMEC-1 (bottom) cell cultures treated with non-targeting (siRNA-NT) or siRNA specific to FBXO31 transcripts (siRNA-FBXO31). Shown are representative images selected from four independent experiments. **(B)** Quantitative analysis of infectious VV released by siRNA-FBXO31 and siRNA-NT treated cultures. Statistics were derived from three independent experiments. **(C)** Quantitative analysis of VV infectious virus in untreated control and SMER3-treated culture. SMER3 was added at 2 hpi and VV infectious titers were determined at 48 hpi by limiting dilution of the conditioned media. Statistics were derived from three independent experiments. Red asterisks in **(B)** and **(C)** indicate statistical significance (Wilcoxon rank-sum test *P*-value ≤ 0.05)

### Role of the SCF E3 ubiquitin ligase complex in VV infectivity

Our RNA-seq and MeRIP-seq surveys revealed differential regulation of genes involved in protein ubiquitination process, a post-translational modification critical for protein turnover. At 24 hpi, several differentially expressed genes were enriched in pathways regulating protein polyubiquitination (GO:0000209; FDR-adjusted *P*-value = 0.13) and protein deubiquitination (GO:0016579; FDR-adjusted *P*-value = 0.16) (Table S2). In parallel, we identified differentially m⁶A-modified transcripts associated with post-translational modification (GO:0036211; FDR-adjusted *P*-value > 0.25) and ubiquitin-dependent protein catabolic process (GO:0006511; FDR-adjusted *P*-value > 0.25) (Text S3) across all three time points. These findings suggest that VV infection alters host protein ubiquitination pathways, on transcriptional and post-transcriptional layers, to potentially facilitate viral replication and enhance pathogenicity—an universal strategy utilized by various virus species including VV [80, 81].

F-box proteins (FBXOs), functioning as substrate-recognition components of the Skp/Cullin/F-box (SCF) E3 ubiquitin ligase complex, play a critical role in antiviral immune responses through ubiquitination-mediated protein degradation [82, 83]. Our MeRIP-seq analysis revealed that both *FBXO31* and *CCNF* (also known as FBXO1) exhibited persistent loss of m⁶A modifications in the coding region throughout all time points (Fig. 2). CCNF, a substrate-recognition subunit of the SCF complex, was previously reported to degrade viral infectivity factor and boost host immunity against human immunodeficiency virus 1 [63]. To date, the role of FBXO31in host-virus interactions has not been defined.

Utilizing RNAi-mediated knockdown of *FBXO31* transcripts (fold change = 8.96E-02; Figure S11) combined with quantitative functional assays (Fig. 5), here we demonstrate that FBXO31 is a host factor that supports VV replication.

Upon depletion of *FBXO31* transcripts with RNAi, we infected HMEC-1 cells with VV and performed immunofluorescence analysis 24 hpi to investigate the impact on virus replication. We observed significant reduction of VV protein expression in both Vero and HMEC-1 cells with *FBXO31* transcript knockdown compared to controls treated with non-targeting siRNA (siRNA-NT) (Fig. 5A). We further evaluated the impact of *FBXO31* knockdown by inoculating naïve Vero cells with supernatants from RNAi treated and VV-infected Vero or HMEC-1 cultures. The result of virus titration assays revealed significant decreases in VV infectious titer production in both *FBXO31*-depleted Vero (fold change = 8.38E-03; Wilcoxon rank-sum test *P*-value = 4.04E-02) and HMEC-1 cultures (fold change = 1.19E-04; Wilcoxon rank-sum test *P*-value = 4.04E-02) (Fig. 5B).

Next, we investigated the role of SCF E3 ubiquitin ligase function in VV infectivity. Treatment of VV infected HMEC-1 cultures with 1 µM SMER3—a selective inhibitor of the SCF^Met30^ E3 ubiquitin ligase [84, 85]—at 2 hpi significantly reduced VV infectious titers at 48 hpi (fold change = 8.78E-03; Wilcoxon rank-sum test *P*-value = 3.83E-02) compared with SMER3-free controls (Fig. 5C). Using a CellTiter-Glo assay, we detected cytotoxicity (i.e., reduced ATP levels) in uninfected Vero cells treated with 1 µM SMER3 for 48 h, whereas no decline in ATP levels was observed in uninfected HMEC-1 cultures (data not shown). Lower SMER3 concentrations (< 1 µM) did not reduce infectious VV titers in either HMEC-1 or Vero cells. Because cytotoxicity prevented testing SMER3 at concentrations known to inhibit SCF^Met30^ E3 ligase activity, we were unable to directly evaluate inhibition of the E3 ligase in Vero cells.

To summarize, we observed a persistent loss of m⁶A modifications in *FBXO31* mRNA during VV infection and found that this adaptor to the Skp/Cullin/F-box (SCF) E3 ubiquitin ligase complex plays important role in the production of infectious particles by both HMEC-1 and Vero cells. Furthermore, pharmacologic inhibition of the SCF E3 ubiquitin ligase attenuated VV infectivity in human endothelial cells, supporting the notion that VV exploits host ubiquitination machinery to facilitate its replication.

### VV transcripts gain m⁶A modifications

**Fig. 6:**
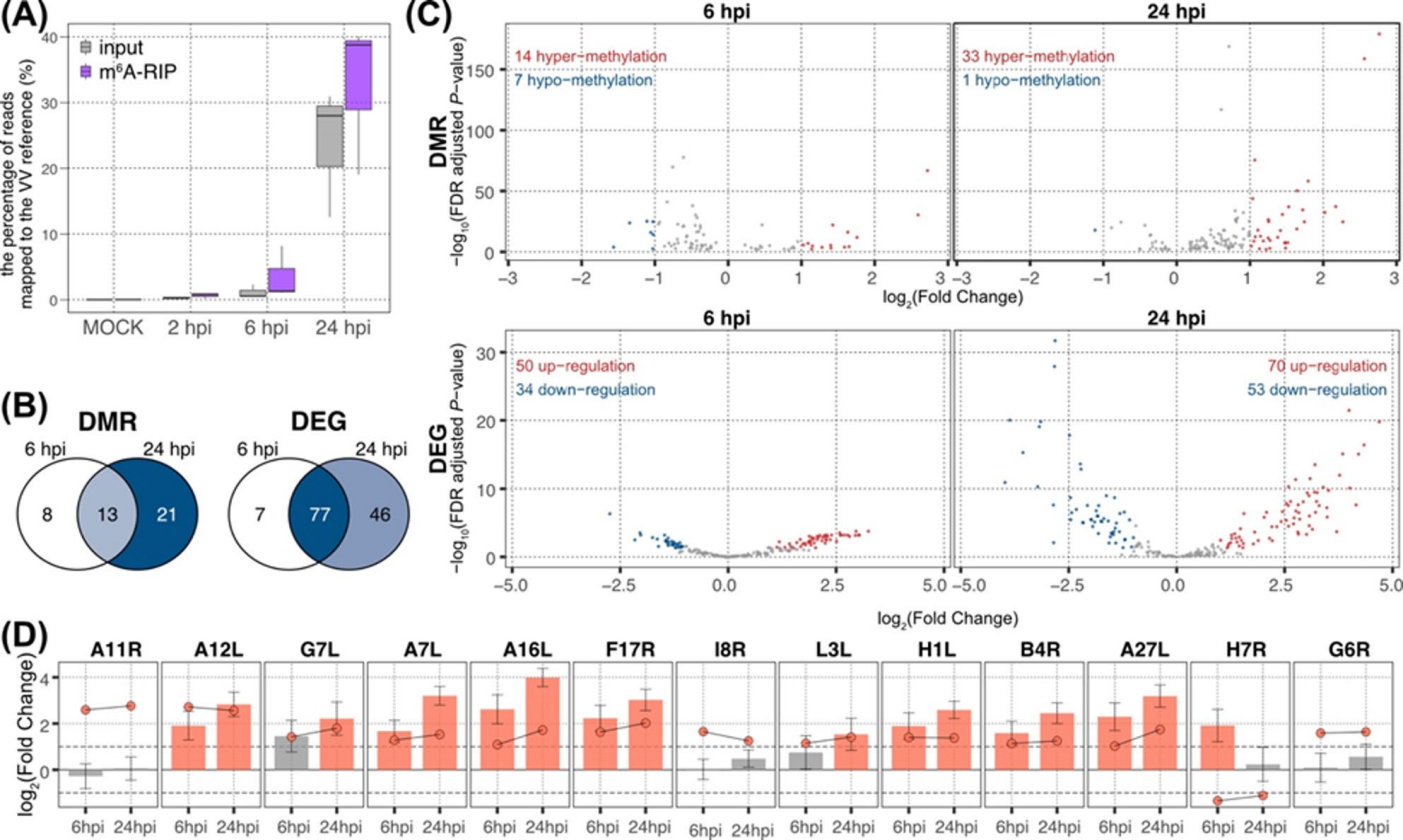
Summary of differential analyses on VV transcriptome and m^6^A epitranscriptome. **(A)** The proportion of total reads mapped against VV and host reference genomes. **(B)** Venn diagrams of VV differentially m^6^A modified regions (top) and VV differentially expressed genes (bottom). **(C)** Scatter plots of VV differentially m^6^A modified regions (top) and VV differentially expressed genes (bottom). Blue and red dots indicate statistically significant changes. **(D)** VV genes with consistent changes in m^6^A modifications between 6 hpi and 24 hpi. Each plot depicts log_2_(fold change) of differential m^6^A modifications (dots) and differential gene expressions (bars). Orange-colored features indicate statistical significance defined by |log_2_(fold change)| ≥ 1 and FDR-adjusted *P*-value ≤ 0.05. Dots connected with lines indicate differential m^6^A modifications detected in the same locus

To investigate the dynamics of VV transcriptome and m⁶A epitranscriptome, we quantified the proportion of reads mapped to the VV-WR reference genome relative to the reads mapped to the combined host and virus reference genomes (VV-WR and *C. sabaeus*). Fitting with the progression of VV infection of Vero cells, the proportion of RNA-seq reads mapped to the VV genome increased from 0.45 ± 0.37% at 2 hpi to 28.21 ± 10.84% at 24 hpi (Fig. 6A). Notably, VV reads were relatively enriched in the m⁶A-RIP fractions compared with their input counterparts (Fig. 6A). While the m⁶A modifications on the host mRNAs were highly dynamic across different time points (Fig. 1D), *de novo* accumulation of m⁶A peaks on the viral transcripts were steady. First, we identified VV DMRs and DEGs at 6 hpi and 24 hpi (|log₂FC| ≥ 1 and FDR-adjusted *P*-value ≤ 0.05), using 2 hpi as the reference sample (Table S5, columns H-I and N-O). Therefore, viral transcripts that were not produced at 2 hpi were omitted from the analysis. Significant changes were defined by |log₂FC| ≥ 1 and FDR- adjusted *P*-value ≤ 0.05. We identified 21 and 34 DMRs at 6 hpi and 24 hpi, respectively (13 DMRs common between two time points), while 84 and 123 DEGs were detected at 6 hpi and 24 hpi, respectively (77 DEGs common between two time points) (Fig. 6B).

Changes in both m⁶A modification and gene expression were highly consistent in the replication cycle course and reflected coordinated, cascade-like expression of viral genes required for maximum efficiency of VV particle formation [86]. Nearly all DMRs at 24 hpi (97.1%) were contributed by gains in m⁶A modifications, while VV DEGs included mixed levels of up- and down-regulated genes (Fig. 6C). Most VV DMRs shared between the 6 and 24 hpi maintained steady accumulation of m^6^A modifications (Fig. 6D). Among the 13 VV transcripts with DMRs only *H7R*—an early VV gene involved in progeny formation [87]—showed consistent loss of m⁶A modifications and increased transcript levels at 6 hpi (Fig. 6D). Similarly, all 77 VV genes identified as DEGs showed consistent expression patterns at 6 and 24 hpi. Specifically, out of 31 VV DEGs inhibited at 6 hpi, 29 transcripts were gradually shut down at the late stage of VV infection, while from 46 VV DEGs activated at 6 hpi, 44 remained up-regulated at 24 hpi (Table S5). These results demonstrate that VV transcripts undergo a coordinated and directional trend of m⁶A modification and gene expression during the virus replication cycle. To capture VV transcripts not detected at 2 hpi, we performed a secondary differential analysis comparing 24 hpi with 6 hpi (Table S5, columns J, P, and V). Thus, we identified 11 additional differentially regulated VV transcripts, including 5 VV DMRs and 6 VV DEGs. Consistent with the progressive accumulation of m⁶A on VV transcripts (Fig. 6C), all VV DMRs resulted from m⁶A accumulation without significant changes in their gene transcription at 24 hpi relative to 6 hpi (Table S5, column P). Four of these VV DMRs are involved in late infection processes, such as host immune suppression and virion formation (*A33R*, *A4L*, *B29R*, and *C23L*). Interestingly, while DEG encoding viral proteins regulating the early stages of VV replication (H5R, H7R, and F11L) were down-regulated, several VV genes associated with virion attachment and inhibition of innate immune responses were up-regulated at 24 hpi relative to 6 hpi, (Table S5, column P). Together, these findings suggest that there is a combination of late-stage virus replication and secondary infection events driven by newly produced virions at 24 hpi.

Consistent with our findings that the host m⁶A machinery is required for efficient VV protein expression (Fig. 4F), the progressive accumulation of m^6^A modifications on VV transcripts further supports the idea that VV exploits the host m⁶A machinery for successful completion of viral life cycle. Furthermore, up-regulation of viral *b4r* transcription and consequent accumulation of m⁶A modifications on the *B4R* mRNA (Fig. 6D), is consistent with our prior findings that Skp/Cullin/F-box (SCF) E3 ubiquitin ligase complex of the host cells is important factor for VV replication (Fig. 5). B4R is a viral F-box protein with multiple Ankyrin domains that hijacks host’s SCF E3 ubiquitin ligase and facilitates virion releases and ability to spread [88].

Although the precise mechanisms of the m⁶A-mediated regulation on VV gene expression remain to be elucidated, our results emphasize the importance of m^6^A epitranscriptome regulation in double-stranded DNA virus replication and host-virus interaction.

## Discussion

In this study, we implemented a temporally resolved, multi-layered analysis of the host and viral gene regulation and m^6^A epitranscriptome dynamics throughout VV infection. Leveraging MeRIP-seq and RNA-seq across three stages of VV infection, we uncovered a dynamic host m⁶A landscape that precedes the transcriptional responses, implicating various aspects of host-virus interactions. Within this framework, we identified novel m⁶A biomarkers associated with infection—including YTHDF1 and FBXO31—that may promote VV infectivity. Supported by multiple quantitative validation assays, our findings highlight early infection signatures in host m^6^A profile that aligns with a model where VV exploits host m⁶A regulation and protein ubiquitination to facilitate its replication.

### Host m⁶A modifications function as early biomarkers of VV infection

A key observation from our MeRIP-seq and RNA-seq survey was the abundance of changes in host m^6^A epitranscriptome shortly upon infection (i.e., 2 and 6 hpi) while changes in host gene expression occur at later stage of virus replication (Figs. 1C and S4). The temporal disconnect between transcriptional and post-transcriptional layers of the host gene expression system indicates that m⁶A functions as an early, sensitive marker of viral infection, enabling VV to exploit host post-transcriptional regulatory mechanisms. This finding also aligns with previous reports that VV suppresses host transcription early upon infection [89, 90], enforcing “host shutoff” before transcriptional feedback occurs. Thus, m⁶A profiling provides critical insights on the temporal axis of host-virus interactions that would have been missed by transcriptomics alone.

### Host factors with differential m^6^A modifications are involved in virus infection

We identified multiple host genes, regulating innate immune responses, with differentially m^6^A-modifed transcripts across different stages of VV replication, including *IRF3*, *ILF3* [48, 50], *CTBP1* [51, 52], *AKT1* [54, 55], *TRIM26* [56, 57], and *RANGAP1* [58] (Text S2). We also observed changes in genes associated with viral infectivity: *HRAS* [45], *EPHA2* [46, 47], *BSDC1* [59], and *CCNF* [63] (Text S2). Most of these genes exhibited loss of the m^6^A mark. However, we found no correlation between m^6^A-modification dynamics and gene transcript levels. Specifically, TRAF2 was hypo-methylated early upon infection and was up-regulated at 24 hpi, while HDAC4 transcripts acquired new m^6^A-marks throughout the virus replication cycle and were similarly up-regulated at 24 hpi (Fig. 2). Both TRAF2 and HDAC4 are key host factors regulating host immune responses to VV infection [65, 68]. VV induces proteasomal degradation of HDAC4 via virulence factor C6L, encoded by the viral genome, as early as 2 hpi [65], which concurs with our finding showing up-regulation of *C6L* at 2 hpi (Table S5). This temporally staggered regulation of viral C6L and host HDAC4 highlights a complex, dynamic interplay that warrants further experimental validation.

### Interplay between m⁶A modification machinery and VV infectivity

Host m⁶A regulation involves a coordinated network of writers, erasers, and readers [91], generating diverse biological outcomes. In line with the importance of m⁶A in viral replication [15] and cellular functions in general [75], we observed a progressive m⁶A enrichment on the VV transcripts (Fig. 6B and C) and a reduced VV protein production in cells with RNAi knockdown of m^6^A-writer METTL3 (Fig. 4F). Among host DMR transcripts, *YTHDF1* stood out for its transient m⁶A gain in the 5′ UTR at 6 hpi. Quantitative assays—including smRNA-FISH, PLA, immunocytochemistry, and proteomics—demonstrated that this m^6^A gain facilitated *YTHDF1* mRNA translation over the course of VV infection. This finding supports a model in which 5′ UTR m⁶A promotes cap-independent translation [77], allowing YTHDF1 protein expression to be compensated for when total transcript levels decline. Such autoregulatory control may extend broadly, as six of eight m⁶A regulators displayed altered m⁶A levels without gene expression changes (Fig. 2).

Importantly, RNAi knockdown of *YTHDF1* transcripts significantly reduced VV protein expression (Fig. 4F), indicating its functional relevance to the viral replication process. As a m^6^A reader, YTHDF1 is known to promote mRNA translation and stability while supporting cell survival at hypoxic stress [92, 93]. Notably, YTHDF1 has been shown to modulate innate immunity by regulating translation of TRAF6 via m^6^A-dependent mechanism [94]. Both our MeRIP-seq and RNA-seq analyses showed that gene expression and m^6^A modification levels of *TRAF6* transcript doubled at 24 hpi (Fig. 2), suggesting that TRAF6 has a role in VV infection. Additionally, we observed similar dynamics of YTHDF1 and ARHGEF2 protein abundances in VV-infected HMEC-1 cells (Table S4 and Figure S9). *ARHGEF2* mRNA is a known target of YTHDF1 [95] and has been implicated in promoting cell survival and transformation [96]. ARHGEF2 also regulates vesicle trafficking through Rho GTPase signaling [97], a process that can facilitate viral particle export. Therefore, YTHDF1 may have an indirect role in virus replication by promoting survival of host cells as factories generating infectious virus particles.

These findings underscore the role of host m⁶A machinery in promoting VV infection. Previously, 3-deazaadenosine (DAA), a methyl donor-depleting agent, was tested as broad-spectrum antiviral therapeutic in animal models [98–100]. Given the essential role of m⁶A machinery in mammalian cell survival, its inhibition poses safety challenges for therapeutic targeting.

### FBXO31 and the SCF E3 ubiquitin ligase complex promote VV infectivity

Host protein ubiquitination machinery is involved in diverse cell regulatory mechanisms by marking proteins for degradation. In this study, we identified several genes encoding RING E3 ubiquitin ligases, *TRAF2*, *TRAF5*, and *TRAF6*, that were up-regulated at 24 hpi (Fig. 2). Although these genes are known to activate the anti-viral, type I IFN pathway [69, 70, 101], they are host factors found to promote vaccinia virus [68] and flavivirus [102] infectivity. Here, we identified *FBXO31* and *CCNF*, two F-box genes with persistently hypo-methylated transcripts during VV infection (Fig. 2). These genes encode components of the SCF E3 ubiquitin ligase complex, acting as adaptors for targeted proteolytic degradation [65, 82]. CCNF has been previously shown to boost host immunity against human immunodeficiency virus [63]. In this study, we demonstrate that FBXO31 supports VV replication (Fig. 5A) and the release of infectious virus titer (Fig. 5B). FBXO31 is known to target Cyclin D1 for degradation and to induce cell cycle arrest [82]. This function aligns with strategy commonly utilized by various virus species, including VV, taking advantage of cell cycle checkpoints for completion of the replication cycle [103–105].

Viruses utilize host E3 ubiquitin ligases to promote replication by inactivation of host antiviral factors [106, 107] or to facilitate viral assembly [108]. The host SCF E3 ligases have been exploited by RNA viruses to degrade APOBEC3G, a crucial antiviral defense factor in host innate immunity [109, 110]. Thus, FBXO31 role in VV replication could stem from its function as an adaptor in the SCF E3 ubiquitin ligase complex. This statement is in agreement with our finding that the viral *B4R* transcript, encoding F-box/ankyrin protein that hijacks host SCF E3 ligases to aid viral spread [88], was both up-regulated and m⁶A hyper-methylated throughout the VV replication cycle (Fig. 6D). Furthermore, we demonstrate that a selective SCF E3 ligase inhibitor, SMER3 significantly reduces VV infectious titers in HMEC-1 cell cultures (Fig. 5C). Taken together, these findings implicate FBXO31 and SCF E3 ligases as host factors that enhance VV infectivity, highlighting their potential as therapeutic targets against poxvirus.

### m⁶A modifications on VV transcripts

The m⁶A accumulation on viral transcripts has been linked to increased infectivity [13, 15, 16, 89–92]. In this study, we observed a steady gain in m⁶A modifications on viral transcripts encoding proteins regulating virus entry, transcription, assembly, and release (Fig. 6D). Coupled with the dynamic changes in the host RNAs m⁶A landscape, induced by the VV infection, and drastic decrease of infectious virus production in host cells with knockdown of the m^6^A writer (METTL3) and reader (YTHDF1) (Figs. 4F and S10), our findings suggest that the m^6^A modifications on VV transcripts are important for the VV gene expression and successful colonization of the host. Altogether, this study positions the host m⁶A machinery as a key driver of VV proliferation.

### Limitations and future directions

While our study provides important insights, its limitations should be noted. First, MeRIP-seq lacks base-level resolution to identify exact m⁶A sites, as it relies on immunoprecipitation of fragmented RNA. Second, we primarily relied on bulk transcript and protein measurements, which may obscure cell-to-cell heterogeneity. Therefore, we clarified the nascent YTHDF1 transcripts and their m^6^A modification status in single-cell resolution with smRNA-FISH and PLA, followed by a direct RNA sequencing on the ONT platform. Third, while we validated the roles of YTHDF1 and FBXO31 in VV infection by RNAi gene knockdown, the exact mechanisms regulating the m⁶A modification status and gene activity requires further investigation.

## Conclusion

In summary, our study reveals that VV benefits from the m^6^A machinery for successful colonization of the host cells. The rapid and widespread remodeling of the host m⁶A landscape highlights the m^6^A modification as biomarker of early stage VV infection. VV exploits host protein ubiquitination pathways, exemplified by FBXO31 and the SCF E3 ligase complex, to further support its proliferation. Together, these findings establish a framework for understanding how VV orchestrates host gene expression regulation layers—across RNA modification, gene expression, and protein expression—to optimize its proliferation, offering new insights into potential therapeutic targets against poxvirus.

## Supplementary Information

Supplementary Material 1.

Supplementary Material 2.

Supplementary Material 3.

Supplementary Material 4.

Supplementary Material 5.

Supplementary Material 6.

Supplementary Material 7.

Supplementary Material 8.

Supplementary Material 9.

Supplementary Material 10.

Supplementary Material 11.

## Data Availability

The raw sequencing data generated in this study are available in NCBI’s Sequence Read Archive (SRA) under BioProject accession number PRJNA1190593 as well as in NCBI’s Gene Expression Omnibus (GEO) under accession number GSE284044.
