# Supplementary material for: Poxvirus infection triggers remodeling of host m⁶A epitranscriptome and benefits from the m⁶A regulatory responses
Source: Virol J. 2026 Apr 11;23:134. doi: 10.1186/s12985-026-03160-y (PMC13202759; doi:10.1186/s12985-026-03160-y)
Supplement: Supplementary file 1 — Supplementary Material 1. [file 12985_2026_3160_MOESM1_ESM.pdf]

Image Report: a-YTHDF1&Markr-2025-09-23 Vero-Mphage

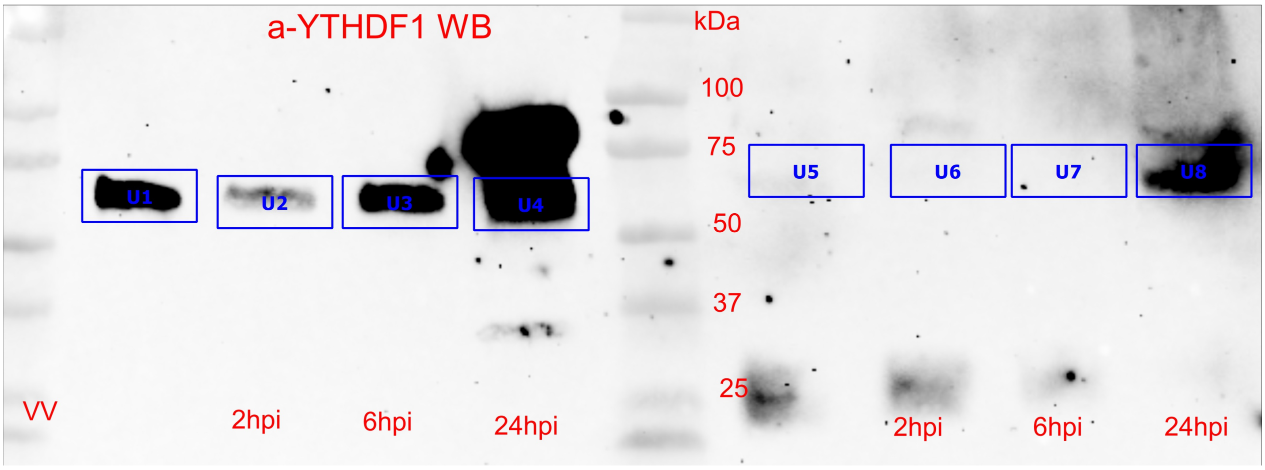

D:\ChemiDoc Images 2025-09-25_18.56.30\a-YTHDF1&Markr-2025-09-23 Vero -Mphage.scn

---

Acquisition Information

Imager	Merged Image

Image Information

Acquisition Date	9/25/2025 5:53:47 PM
User Name	229740
Image Area (mm)	X: 79.1 Y: 28.9
Pixel Size (µm)	X: 131.0 Y: 130.9
Data Range (Int)	381 - 37890

Notes

Merged from:
Image 1: sofiya 2025-09-23 Marker
Image 2: a-YTHDF1_ 2025-09-23 Vero

Use the merged image to estimate molecular weight only if sample was not moved between acquisition of individual images.

Analysis Settings

Volume Analysis	Background subtraction method: Local<br>Quantity regression method: Linear

Volume Analysis

No.	Label	Type	Volume (Int)	Adj. Vol. (Int)	Mean Bkgd. (Int)	Abs. Quant.	Rel. Quant.	# of Pixels	Min. Value (Int)	Max. Value (Int)	Mean Value (Int)	Std. Dev.	Area (mm2)
1	U1	Unknown	22,947,010	18,645,658	3,128.3	N/A	N/A	1,375	2,577	35,983	16,688.7	14,761.1	23.6

2	U2	Unknown	9,929,166	5,626,640	3,129.1	N/A	N/A	1,375	2,546	30,746	7,221.2	5,586.1	23.6
3	U3	Unknown	24,640,184	15,541,952	6,616.9	N/A	N/A	1,375	2,767	36,341	17,920.1	14,014.6	23.6
4	U4	Unknown	36,341,670	16,850,861	14,175.1	N/A	N/A	1,375	3,040	36,285	26,430.3	12,738.3	23.6
5	U5	Unknown	6,946,505	391,989	4,766.9	N/A	N/A	1,375	3,619	36,449	5,052.0	1,553.1	23.6
6	U6	Unknown	6,096,596	66,567	4,385.5	N/A	N/A	1,375	3,657	5,339	4,433.9	325.4	23.6
7	U7	Unknown	6,968,546	-58,710	5,110.7	N/A	N/A	1,375	3,796	36,592	5,068.0	2,078.9	23.6
8	U8	Unknown	38,219,310	13,740,973	17,802.4	N/A	N/A	1,375	6,214	36,626	27,795.9	8,619.5	23.6
