# Supplementary material for: Poxvirus infection triggers remodeling of host m⁶A epitranscriptome and benefits from the m⁶A regulatory responses
Source: Virol J. 2026 Apr 11;23:134. doi: 10.1186/s12985-026-03160-y (PMC13202759; doi:10.1186/s12985-026-03160-y)
Supplement: Supplementary file 2 — Supplementary Material 2. [file 12985_2026_3160_MOESM2_ESM.pdf]

Image Report: a-ActB&Marker2025-09-25-HMEC-Mphage

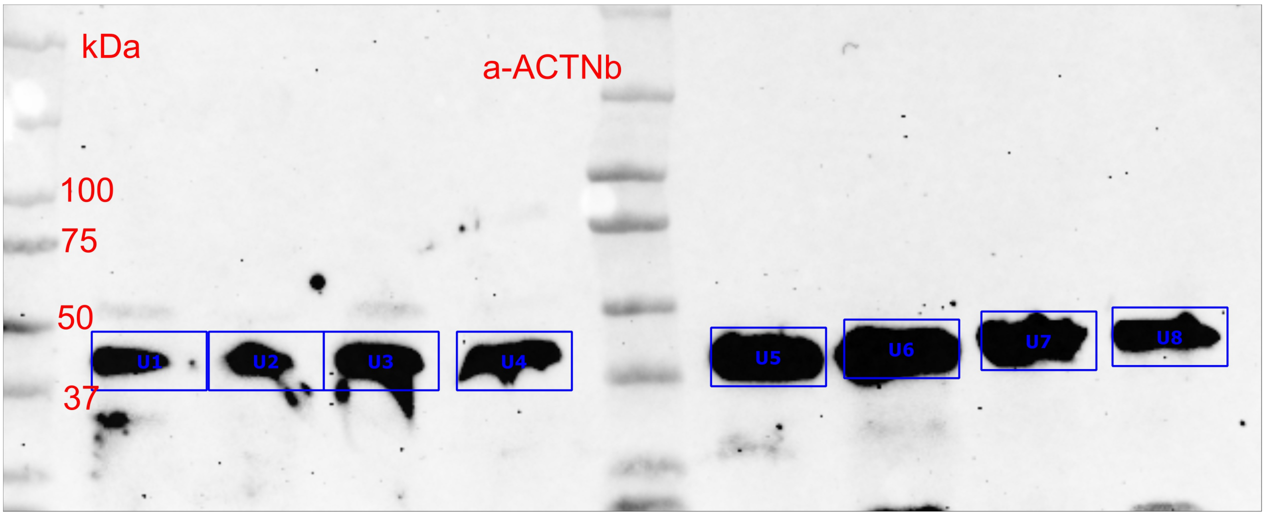

D:\ChemiDoc Images 2025-09-25_18.55.55\a-ActB&Marker2025-09-25-HMEC -Mphage.scn

Acquisition Information

Imager	Merged Image

Image Information

Acquisition Date	9/25/2025 5:19:21 PM
User Name	229740
Image Area (mm)	X: 81.7 Y: 32.9
Pixel Size (µm)	X: 131.0 Y: 131.0
Data Range (Int)	152 - 36320

Notes

Merged from:
Image 1: a-ActB 2025-09-25 HMEC-Mphage
Image 2: Marker 2025-09-25 18h45m48s

Use the merged image to estimate molecular weight only if sample was not moved between acquisition of individual images.

Analysis Settings

Volume Analysis	Background subtraction method: Local<br>Quantity regression method: Linear

Volume Analysis

No.	Label	Type	Volume (Int)	Adj. Vol. (Int)	Mean Bkgd. (Int)	Abs. Quant.	Rel. Quant.	# of Pixels	Min. Value (Int)	Max. Value (Int)	Mean Value (Int)	Std. Dev.	Area (mm2)

1	U1	Unknown	20,478,124	15,228,957	3,175.5	N/A	N/A	1,653	1,871	34,874	12,388.5	13,308.8	28.4
2	U2	Unknown	21,980,571	14,282,625	4,657.0	N/A	N/A	1,653	1,774	34,946	13,297.4	13,334.1	28.4
3	U3	Unknown	33,069,831	22,226,865	6,559.6	N/A	N/A	1,653	1,881	35,196	20,005.9	14,679.4	28.4
4	U4	Unknown	29,980,848	25,277,509	2,845.3	N/A	N/A	1,653	2,051	35,365	18,137.2	14,652.6	28.4
5	U5	Unknown	46,215,162	34,509,433	7,081.5	N/A	N/A	1,653	2,707	35,601	27,958.4	11,438.6	28.4
6	U6	Unknown	49,641,865	27,009,205	13,691.9	N/A	N/A	1,653	2,586	35,876	30,031.4	10,557.4	28.4
7	U7	Unknown	41,163,580	30,989,853	6,154.7	N/A	N/A	1,653	2,009	35,990	24,902.3	13,905.1	28.4
8	U8	Unknown	30,970,565	24,778,671	3,745.9	N/A	N/A	1,653	1,753	35,548	18,736.0	14,972.8	28.4
