# Supplementary material for: Poxvirus infection triggers remodeling of host m⁶A epitranscriptome and benefits from the m⁶A regulatory responses
Source: Virol J. 2026 Apr 11;23:134. doi: 10.1186/s12985-026-03160-y (PMC13202759; doi:10.1186/s12985-026-03160-y)
Supplement: Supplementary file 3 — Supplementary Material 3. [file 12985_2026_3160_MOESM3_ESM.pdf]

Image Report: a-VV&Markr 2025-09-24HMEC1-Mphage

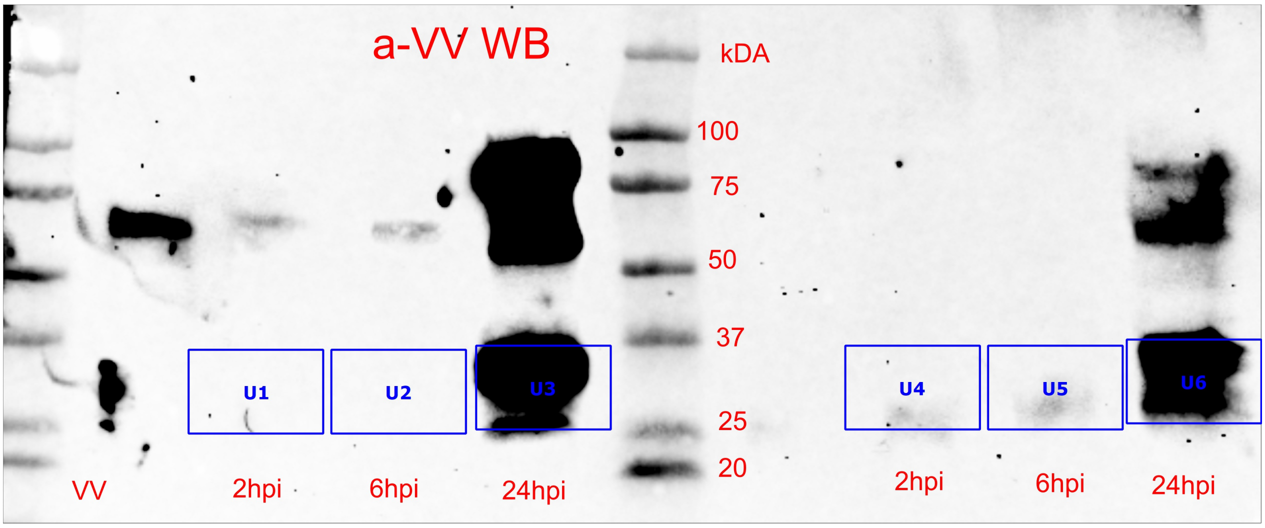

D:\ChemiDoc Images 2025-09-25_18.56.12\a-VV&Markr 2025-09-24-HMEC1-Mphage.scn

Acquisition Information

Imager	Merged Image

Image Information

Acquisition Date	9/25/2025 5:35:03 PM
User Name	229740
Image Area (mm)	X: 80.8 Y: 33.3
Pixel Size (µm)	X: 131.0 Y: 131.0
Data Range (Int)	0 - 50490

Notes

Merged from:
Image 1: a-Vv- 2025-09-24HMEC1-Mphage
Image 2: Marker 2025-09-24 18h08m07s

Use the merged image to estimate molecular weight only if sample was not moved between acquisition of individual images.

Analysis Settings

Volume Analysis	Background subtraction method: Local<br>Quantity regression method: Linear

Volume Analysis

No.	Label	Type	Volume (Int)	Adj. Vol. (Int)	Mean Bkgd.	Abs. Quant.	Rel. Quant.	# of Pixels	Min. Value	Max. Value	Mean Value	Std. Dev.	Area (mm2)

					(Int)				(Int)	(Int)	(Int)		
1	U1	Unknown	4,227,478	423,289	1,405.8	N/A	N/A	2,706	883	29,924	1,562.3	1,127.5	46.4
2	U2	Unknown	4,376,181	59,515	1,595.2	N/A	N/A	2,706	915	2,517	1,617.2	213.8	46.4
3	U3	Unknown	67,287,536	27,626,128	14,656.8	N/A	N/A	2,706	1,275	35,316	24,866.1	13,700.9	46.4
4	U4	Unknown	7,817,143	574,497	2,676.5	N/A	N/A	2,706	1,407	35,200	2,888.8	1,425.0	46.4
5	U5	Unknown	8,555,883	2,403,395	2,273.6	N/A	N/A	2,706	1,445	11,600	3,161.8	1,565.2	46.4
6	U6	Unknown	62,034,503	30,085,148	11,806.9	N/A	N/A	2,706	1,272	35,669	22,924.8	13,546.6	46.4
