# Supplementary figures and images for: Poxvirus infection triggers remodeling of host m⁶A epitranscriptome and benefits from the m⁶A regulatory responses
Source: Virol J. 2026 Apr 11;23:134. doi: 10.1186/s12985-026-03160-y (PMC13202759; doi:10.1186/s12985-026-03160-y)

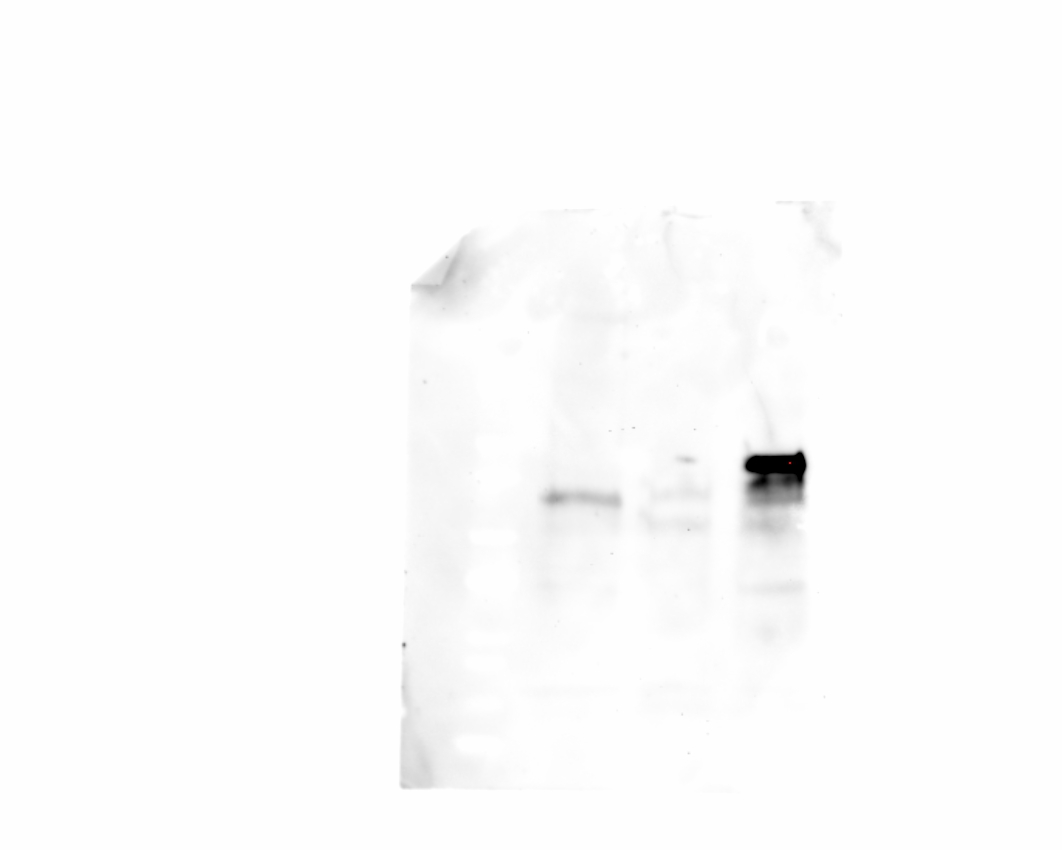

Supplement: Supplementary file 4 — Supplementary Material 4. [file 12985_2026_3160_MOESM4_ESM.jpg]
