# Supplementary material for: Poxvirus infection triggers remodeling of host m⁶A epitranscriptome and benefits from the m⁶A regulatory responses
Source: Virol J. 2026 Apr 11;23:134. doi: 10.1186/s12985-026-03160-y (PMC13202759; doi:10.1186/s12985-026-03160-y)
Supplement: Supplementary file 5 — Supplementary Material 5. [file 12985_2026_3160_MOESM5_ESM.pdf]

Image Report: a-ActB 2025-09-25 Vero-HMECsiR_M3-Y1

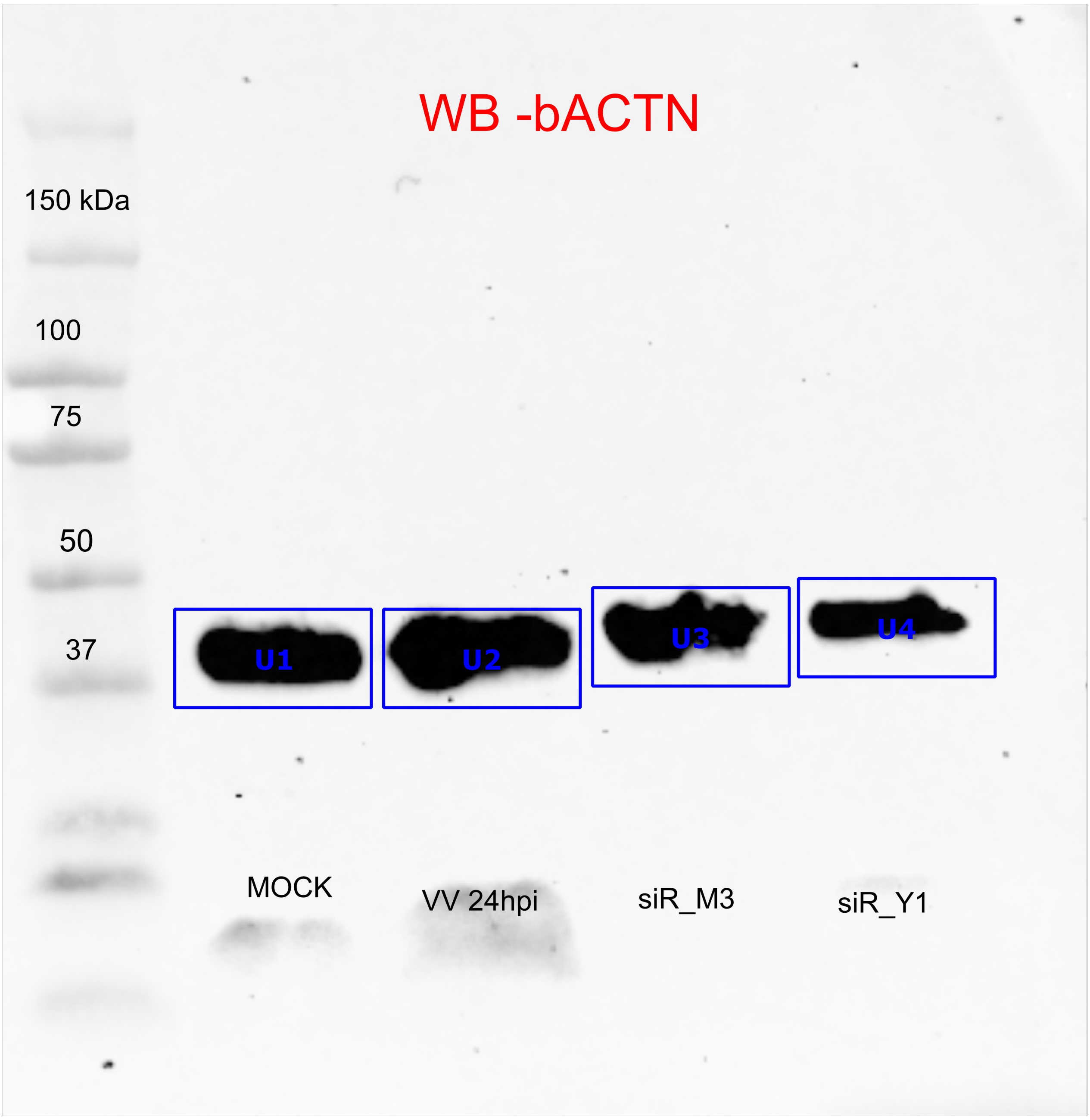

D:\YTHDF1-Images 2025-11-10_Vero-VV\a-ActB 2025-09-25 Vero-HMECsiR_M3-Y1.scn

---

Acquisition Information

Imager	Merged Image

Image Information

Acquisition Date	12/5/2025 4:29:18 PM
User Name	229740

Image Area (mm)	X: 46.2 Y: 47.4
Pixel Size (μm)	X: 131.0 Y: 131.0
Data Range (Int)	367 - 37200

Notes

Merged from:
Image 1: a-ActB 2025-09-25 Vero-HMEC
Image 2: sofiya 2025-09-25 18h45m48s

Use the merged image to estimate molecular weight only if sample was not moved between acquisition of individual images.

Analysis Settings

Volume Analysis	Background subtraction method: Local<br>Quantity regression method: Linear

Volume Analysis

No.	Label	Type	Volume (Int)	Adj. Vol. (Int)	Mean Bkgd. (Int)	Abs. Quant.	Rel. Quant.	# of Pixels	Min. Value (Int)	Max. Value (Int)	Mean Value (Int)	Std. Dev.	Area (mm2)
1	U1	Unknown	39,881,743	32,525,818	3,591.8	N/A	N/A	2,048	3,109	36,879	19,473.5	14,863.4	35.1
2	U2	Unknown	47,385,326	37,939,145	4,612.4	N/A	N/A	2,048	3,110	37,159	23,137.4	14,435.3	35.1
3	U3	Unknown	36,666,660	28,857,939	3,812.9	N/A	N/A	2,048	2,938	37,200	17,903.6	14,839.5	35.1
4	U4	Unknown	27,154,872	20,213,563	3,389.3	N/A	N/A	2,048	2,676	36,825	13,259.2	13,907.9	35.1
