# Supplementary material for: Poxvirus infection triggers remodeling of host m⁶A epitranscriptome and benefits from the m⁶A regulatory responses
Source: Virol J. 2026 Apr 11;23:134. doi: 10.1186/s12985-026-03160-y (PMC13202759; doi:10.1186/s12985-026-03160-y)
Supplement: Supplementary file 6 — Supplementary Material 6. [file 12985_2026_3160_MOESM6_ESM.pdf]

Image Report: a_VV_2025-04-28 siR_M3-Y1

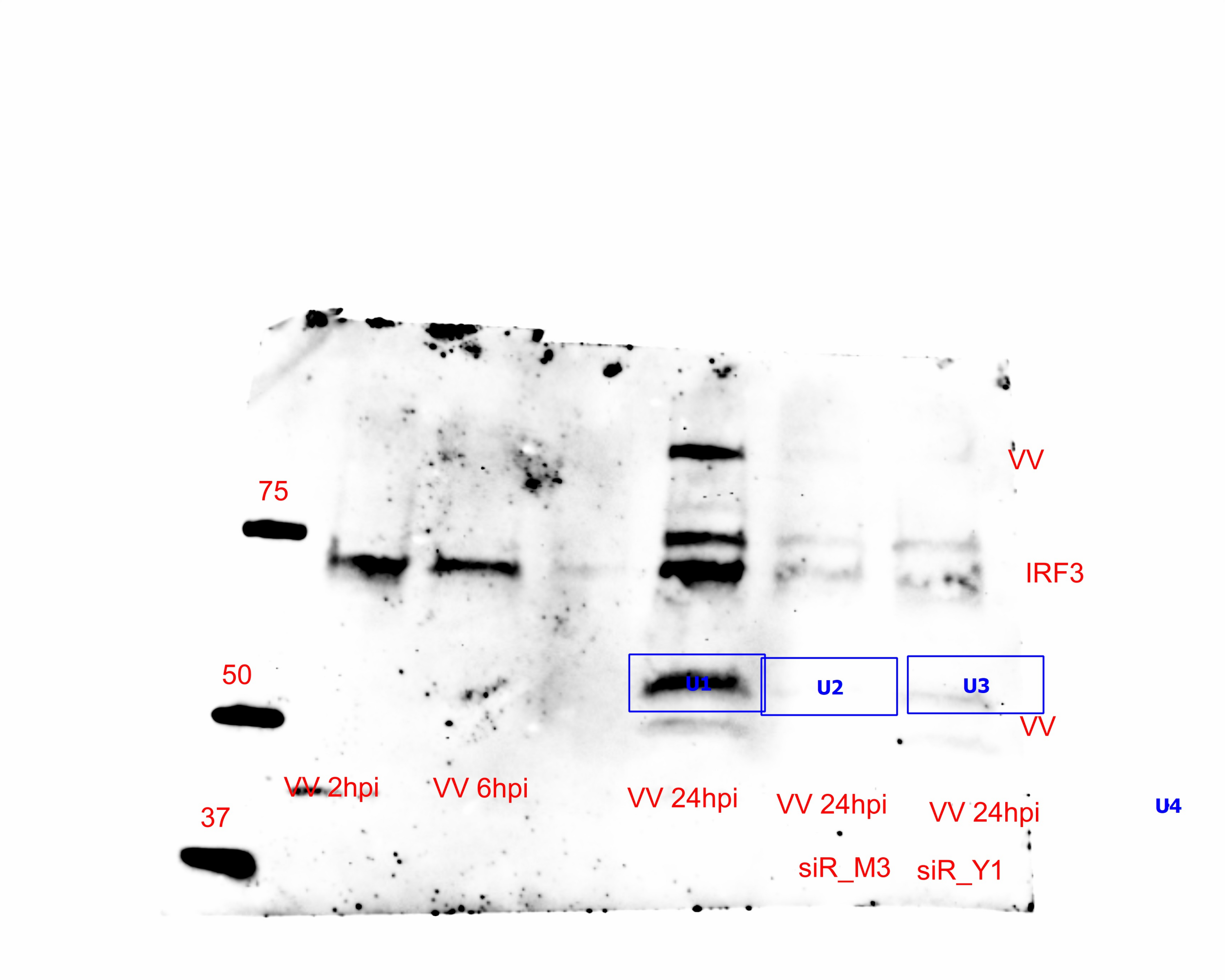

D:\ChemiDoc Images 2025-09-15_17.47.25\sofiya 2025-04-28 20h15m52s\a_VV_2025-04-28 siR_M3-Y1.scn

Acquisition Information

Imager	ChemiDoc™ MP
Exposure Time (sec)	46.666 (Signal Accumulation)
Serial Number	734BR-3916
Software Version	2.4.0.03
Application	Chemiluminescence
Excitation Source	No Illumination
Emission Filter	647SP Filter
Binning	4x4

Image Information

Acquisition Date	4/28/2025 8:16:29 PM
User Name	sofiya

Image Area (mm)	X: 90.0 Y: 72.1
Pixel Size (µm)	X: 131.0 Y: 131.0
Data Range (Int)	502 - 65535

Analysis Settings

Volume Analysis	Background subtraction method: Local<br>Quantity regression method: Linear

Volume Analysis

No.	Label	Type	Volume (Int)	Adj. Vol. (Int)	Mean Bkgd. (Int)	Abs. Quant.	Rel. Quant.	# of Pixels	Min. Value (Int)	Max. Value (Int)	Mean Value (Int)	Std. Dev.	Area (mm2)
1	U1	Unknown	60,840,893	8,608,221	21,477.3	N/A	N/A	2,432	19,857	35,655	25,016.8	3,871.5	41.7
2	U2	Unknown	42,470,334	-932,299	17,846.5	N/A	N/A	2,432	13,854	23,639	17,463.1	2,201.2	41.7
3	U3	Unknown	40,624,087	6,792,845	13,910.9	N/A	N/A	2,432	711	38,558	16,704.0	6,410.2	41.7
4	U4	Unknown	578	-88	665.6	N/A	N/A	1	578	0	578.0	0.0	0.0
