# Supplementary material for: Poxvirus infection triggers remodeling of host m⁶A epitranscriptome and benefits from the m⁶A regulatory responses
Source: Virol J. 2026 Apr 11;23:134. doi: 10.1186/s12985-026-03160-y (PMC13202759; doi:10.1186/s12985-026-03160-y)
Supplement: Supplementary file 8 — Supplementary Material 8. [file 12985_2026_3160_MOESM8_ESM.pdf]

Image Report: sofiya 2025-04-29 19h55m11s+a-ACTNb 2025-04-29-siR-M3-Y

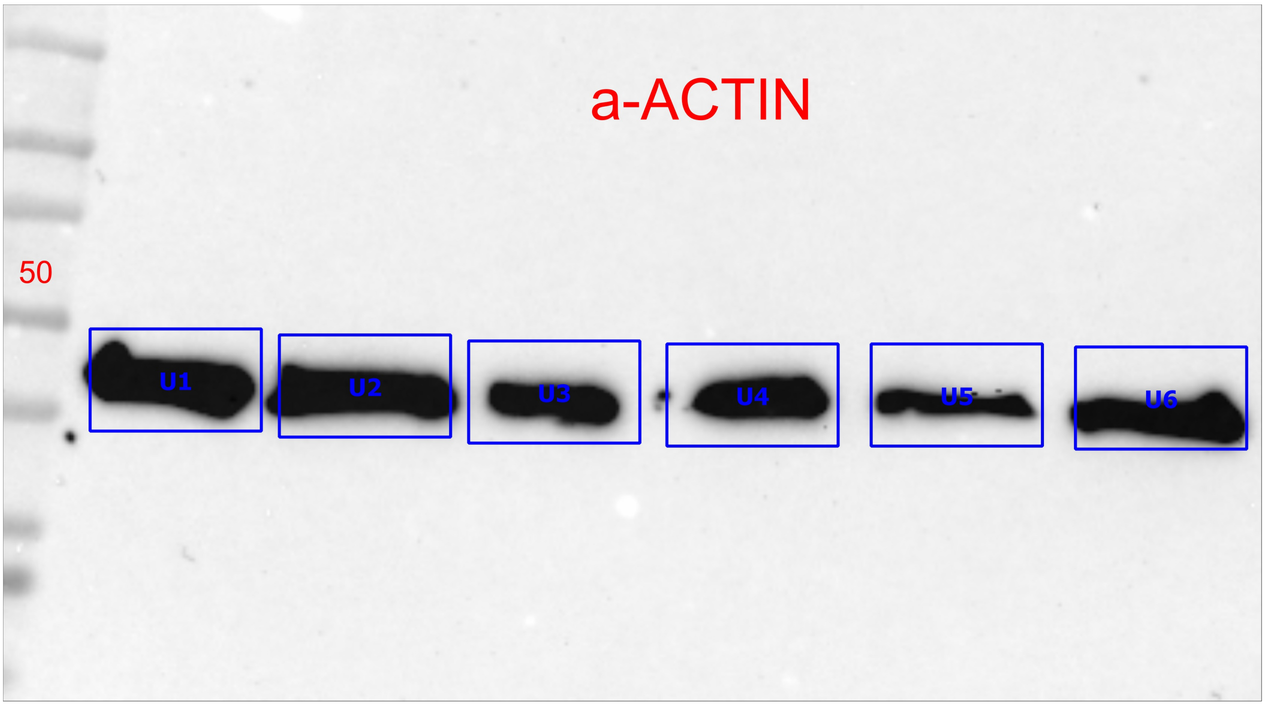

Acquisition Information

Imager	Merged Image

Image Information

Acquisition Date	9/9/2025 11:05:28 AM
User Name	229740
Image Area (mm)	X: 54.9 Y: 30.4
Pixel Size (µm)	X: 131.0 Y: 130.9
Data Range (Int)	3395 - 36205

Notes

Merged from:
Image 1: sofiya 2025-04-29 19h55m11s
Image 2: a-ACTNb 2025-04-29-siR-M3-Y

Use the merged image to estimate molecular weight only if sample was not moved between acquisition of individual images.

Analysis Settings

Volume Analysis	Background subtraction method: Local

	Quantity regression method: Linear

Volume Analysis

No.	Label	Type	Volume (Int)	Adj. Vol. (Int)	Mean Bkgd. (Int)	Abs. Quant.	Rel. Quant.	# of Pixels	Min. Value (Int)	Max. Value (Int)	Mean Value (Int)	Std. Dev.	Area (mm2)
1	U1	Unknown	41,845,735	24,943,874	8,721.3	N/A	N/A	1,938	5,530	36,205	21,592.2	12,206.9	33.2
2	U2	Unknown	40,965,147	20,083,353	10,774.9	N/A	N/A	1,938	5,713	35,067	21,137.8	11,910.1	33.2
3	U3	Unknown	30,411,659	17,720,593	6,548.5	N/A	N/A	1,938	5,542	34,810	15,692.3	11,253.2	33.2
4	U4	Unknown	32,704,819	18,878,867	7,134.1	N/A	N/A	1,938	5,507	35,543	16,875.6	11,631.0	33.2
5	U5	Unknown	25,971,897	13,502,659	6,434.1	N/A	N/A	1,938	5,113	35,272	13,401.4	10,379.9	33.2
6	U6	Unknown	36,133,364	18,388,723	9,156.2	N/A	N/A	1,938	5,681	35,564	18,644.7	12,406.4	33.2
