# Supplementary material for: Poxvirus infection triggers remodeling of host m⁶A epitranscriptome and benefits from the m⁶A regulatory responses
Source: Virol J. 2026 Apr 11;23:134. doi: 10.1186/s12985-026-03160-y (PMC13202759; doi:10.1186/s12985-026-03160-y)
Supplement: Supplementary file 9 — Supplementary Material 9. [file 12985_2026_3160_MOESM9_ESM.pdf]

Image Report: a-YTDHF1_ 2025-06-26 HMEC_VV_siR_Y

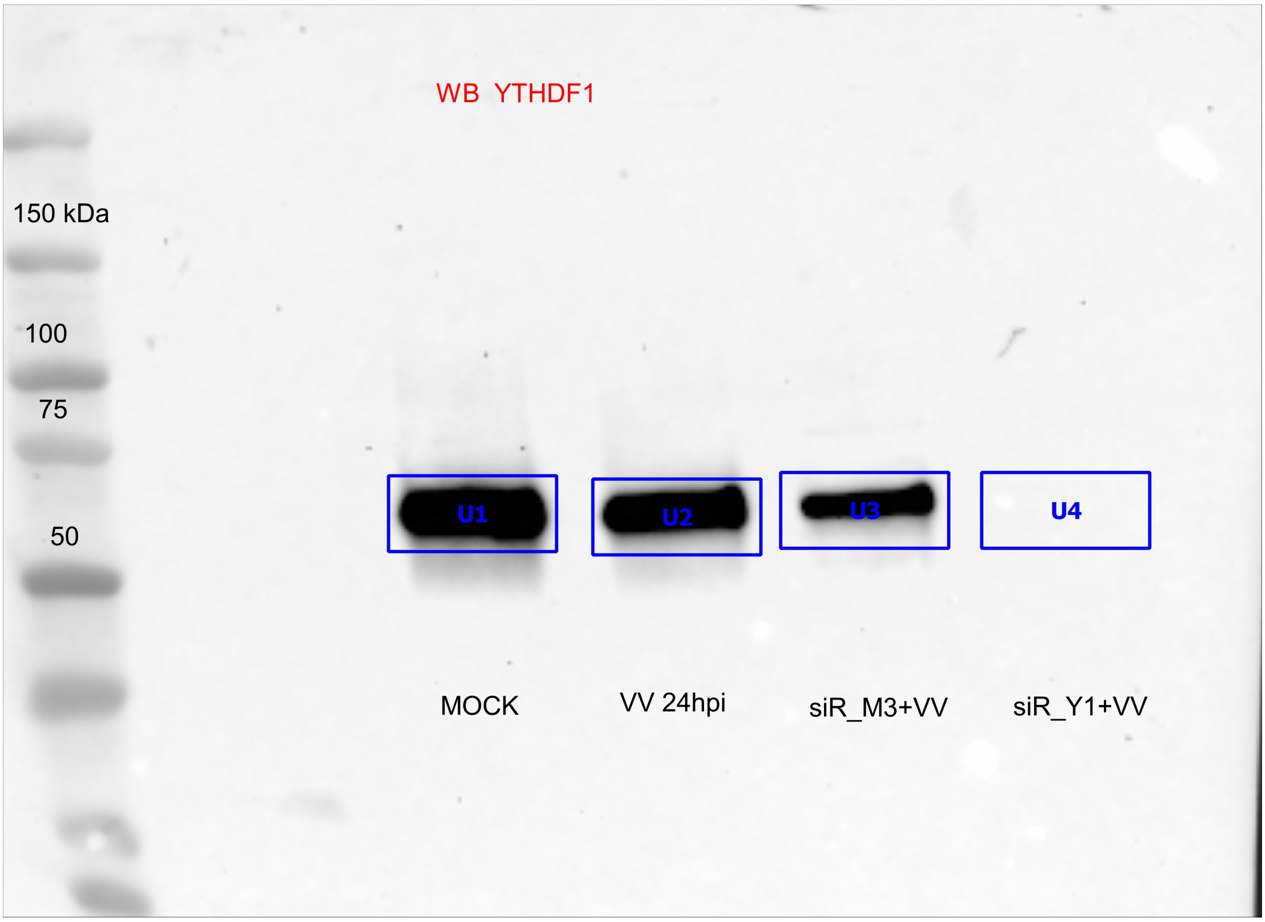

D:\YTHDF1-Images 2025-11-10_Vero-VV\a-YTDHF1_ 2025-06-26 HMEC_VV_siR_Y.scn

Acquisition Information

Imager	Merged Image

Image Information

Acquisition Date	12/5/2025 4:16:14 PM
User Name	229740
Image Area (mm)	X: 50.0 Y: 36.3
Pixel Size (µm)	X: 131.0 Y: 131.0
Data Range (Int)	0 - 34785

Notes

Merged from:
Image 1: a-YTDHF1_ 2025-06-26 HMEC_VV
Image 2: sofiya 2025-06-26 21h24m47s

Use the merged image to estimate molecular weight only if sample was not moved between acquisition

of individual images.

Analysis Settings

Volume Analysis	Background subtraction method: Local<br>Quantity regression method: Linear

Volume Analysis

No.	Label	Type	Volume (Int)	Adj. Vol. (Int)	Mean Bkgd. (Int)	Abs. Quant.	Rel. Quant.	# of Pixels	Min. Value (Int)	Max. Value (Int)	Mean Value (Int)	Std. Dev.	Area (mm2)
1	U1	Unknown	28,267,585	20,995,132	6,199.9	N/A	N/A	1,173	1,384	34,701	24,098.5	11,737.5	20.1
2	U2	Unknown	23,350,724	18,788,086	3,889.7	N/A	N/A	1,173	1,346	34,785	19,906.8	12,999.3	20.1
3	U3	Unknown	15,614,517	13,150,090	2,101.0	N/A	N/A	1,173	1,116	34,522	13,311.6	12,846.2	20.1
4	U4	Unknown	1,493,694	-38,012	1,305.8	N/A	N/A	1,173	0	2,077	1,273.4	204.7	20.1
