# Supplementary material for: Poxvirus infection triggers remodeling of host m⁶A epitranscriptome and benefits from the m⁶A regulatory responses
Source: Virol J. 2026 Apr 11;23:134. doi: 10.1186/s12985-026-03160-y (PMC13202759; doi:10.1186/s12985-026-03160-y)
Supplement: Supplementary file 10 — Supplementary Material 10. [file 12985_2026_3160_MOESM10_ESM.docx]

**Supplemental Materials**

**Poxvirus Infection Triggers Remodeling of Host m⁶A Epitranscriptome and Benefits from the m^6^A Regulatory Responses**

Taehyung Kwon^1^, Demosthenes P. Morales ^2^, Abigale S. Mikolitis^1^, Phillip M. Mach^1^, Cheryl D. Gleasner^3^, Sofiya N. Micheva-Viteva^1^*

^1^ Biochemistry and Biotechnology Group (B-TEK), Bioscience Division, Los Alamos National Laboratory, New Mexico, USA

^2^ Center for Integrated Nanotechnologies (MPA-CINT), Los Alamos National Laboratory, New Mexico, USA,

^3^ Genomics and Bioanalytics Group (B-GEN), Bioscience Division, Los Alamos National Laboratory, New Mexico, USA

* Corresponding author

E-mail address: [sviteva@lanl.gov](mailto:sviteva@lanl.gov)

**Supplemental Texts: Text S1 – S3**

Text S1. Motif analysis of m⁶A sites across differentially m^6^A-modified regions.

Text S2. Persistent differentially m^6^A-modified regions associated with host-virus interaction.

Text S3. Gene set enrichment analysis of GO terms for differentially m^6^A-modified genes.

**Supplemental Figures: Figures S1 – S11**

Figure S1. Summary of host differentially m^6^A-modified regions by locus type.

Figure S2. Summary of the motif analysis of host differentially m^6^A-modified regions.

Figure S3. Dynamics of m^6^A-modified transcripts in VV-infected Vero cells**.**

Figure S4. Dispersion plot of host differential gene expressions and host differential m^6^A modifications.

Figure S5. Changes in m^6^A-modifications detected in *YTHDF1* 5’UTR region.

Figure S6. RT-qPCR analysis for *YTHDF1* transcripts in VV-infected Vero cells.

Figure S7. Fluctuations of *YTHDF1* transcripts in VV-infected cells detected by single molecule RNA fluorescence *in situ* hybridization (smRNA-FISH).

Figure S8. Vaccinia virus spread within monolayers of Vero cells**.**

Figure S9. Label-free MS/MS proteomics analysis of VV-infected human skin endothelial cells (HMEC-1) reveal co-expression of YTHDF1 and ARHGEF2.

Figure S10. RNAi knockdown of *YTHDF1* restricts Vaccinia virus replication in human skin endothelial cells.

Figure S11. Real-Time quantitative PCR evaluation of FBXO31 transcript knockdown by RNAi.

**Text S1. Motif analysis of m⁶A sites across differentially m^6^A-modified regions.**

Motif enrichment analysis identified GACGA as the most enriched 5-mer motif at predicted m⁶A modification sites across all three time points (**Figure S2**). This motif contains the canonical RA*C sequence (R*=*A or G; A*=m⁶A modification site), which partially overlaps with the previously reported DRA*CH motif (D=A, G, or U; H=A, C, or U) [1].

**Text S2. Persistent differentially m^6^A-modified regions associated with host-virus interaction.**

*Early infection phase*: We found *HYAL2*, a known surface receptor for the Jaagsiekte sheep retrovirus [2]; *HRAS*, which encodes a GTPase that facilitates viral entry for hepatitis C and influenza viruses [3]; and *EPHA2*, which promotes entry of various viruses [4, 5]. Additionally, two hypo-methylated DMGs—*ILF3*, encoding interleukin enhancer-binding factor 3, and *IRF3*, encoding interferon regulatory factor 3—are known to mediate host immune responses that restrict viral replication [6-8]. Although *ILF3* overexpression has been reported to suppress innate immune responses in some contexts [8], its transcript level was not significantly altered in our study. Lastly, *CTBP1*, a transcriptional co-repressor known to inhibit viral replication [9, 10], was hypo-methylated during the early phase of infection.

*Late infection phase*: *UBE2M*, known to enhance antiviral activity against RNA viruses [11], displayed hyper-methylation during the late infection phase. In contrast, *AKT1*, which participates in host responses to viral replication [12, 13], and *TRIM26*, which facilitates the replication of hepatitis and herpes simplex viruses [14, 15], were both consistently hypo-methylated.

*Persistent throughout infection*: Three genes showed consistent hypo-methylation: *RANGAP1*, which is essential for influenza virus replication [16]; *BSDC1*, which contains bipartite tryptophan motifs and contributes to VV virion transport [17]; and *CCNF* (also known as *FBXO1*), which restricts virion infectivity through ubiquitination of HIV virulence factors [18]. Two genes were consistently hyper-methylated across the infection period. These included *CREB1*, which regulates host antiviral responses against various viruses [19-21] and *HDAC4*, a key component of type I interferon (IFN) signaling and a known restriction factor for VV infection [22, 23].

**Text S3. Gene set enrichment analysis of GO terms for differentially m^6^A-modified genes.**

GSEA of GO terms revealed that hyper-methylated DMGs at 2 hpi were enriched in genes involved in apoptotic signaling regulation (GO:2001242; FDR-adjusted *P*-value=0.12), as well as genes associated with cell growth and response to growth factor stimulus (GO:0070848 and GO:0071363; FDR-adjusted *P*-value > 0.25). These results suggest that the early stage of VV infection may activate innate immune responses and cell cycle regulatory pathways. In contrast, DMRs identified at 6 hpi and 24 hpi did not show significant enrichment in GO terms or KEGG pathways, and overall GSEA results for these time points yielded low statistical significance (FDR-adjusted *P*-value > 0.25).

The DMGs detected in the early infection phase were enriched in negative of DNA transcription and RNA biosynthesis (GO:0045892 and GO:1902679; FDR-adjusted *P*-value=0.14). Consistently hypo-methylated genes detected in the early infection phase (*BRMS1*, *ILF3*, *HRAS*, *KLF1*, *SRA1*, and *ZNF516*) and across all time points (*ZNF133*, *ZNF496*, and *ZNF671*) were associated with RNA biosynthesis regulation process (GO:2001141) (**Figure 2**).

Several DMGs associated with protein modification process (GO:0036211; FDR-adjusted *P*-value > 0.25) and ubiquitin-dependent protein catabolic process (GO:0006511; FDR-adjusted *P*-value > 0.25). These included 11 DMGs observed during the early infection phase (*B4GAT1, BRMS1, CSNK1G3, CTCF, HMG20B, HRAS, HYAL2, ILF3, STK25, TADA3,* and *TNFSF15*), two DMGs during the late infection phase (*GATAD2A* and *UBE2M*), and six persistent DMGs (*ATXN7L3*, *CREB1*, *FBXO31*, *HDAC4*, *IRF2BP1*, and *RANGAP1*) (**Table S1**). Additionally, we identified five DMGs during the late phase of infection that were associated with the ubiquitin-dependent protein catabolic process (GO:0006511; FDR-adjusted *P*-value > 0.25): *AKT1, ERCC8, FBXO38, LOC103223312,* and *STUB1* (**Table S1**).

**Figure S1. Summary of host differentially m^6^A-modified regions by locus type.**

**
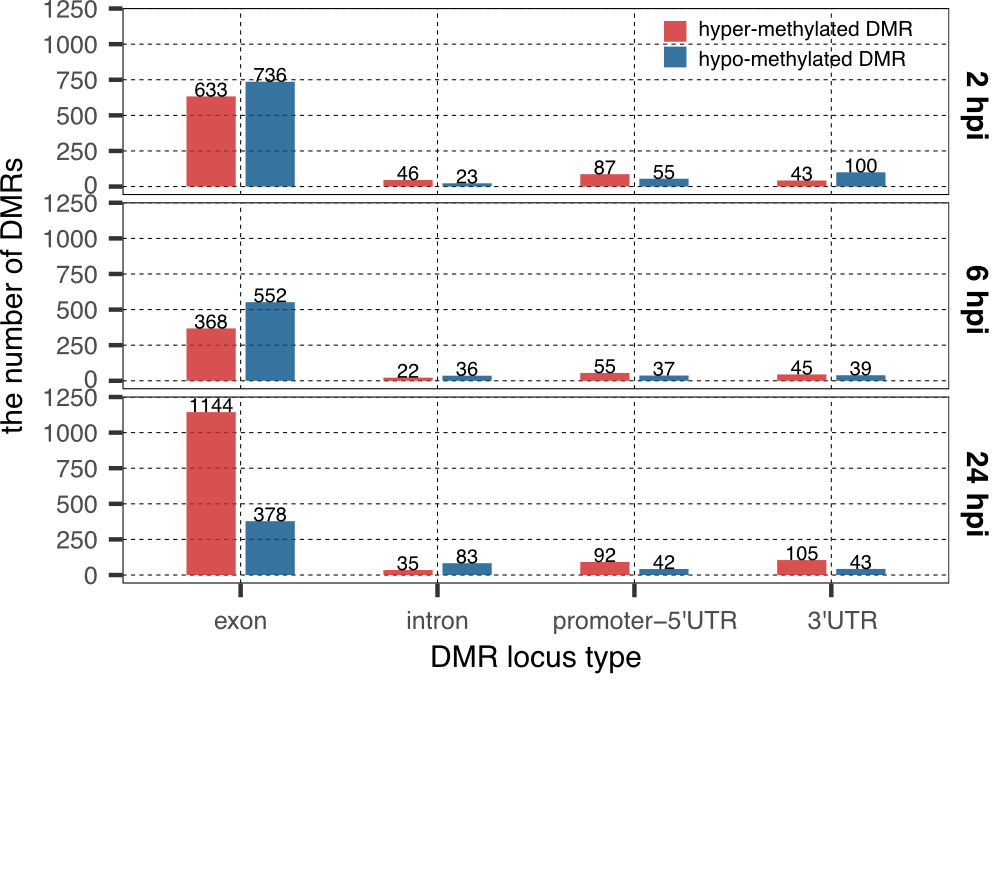
**

**Figure S2. Summary of the motif analysis of host differentially m^6^A-modified regions.**

**Figure S3. Dynamics of m^6^A-modified transcripts in VV-infected Vero cells. (A)** *In situ* fluorescence immunocytochemistry imaging of m^6^A-modifications on global transcriptome (left) and its quantitative analysis (right) The m^6^A-modified transcripts (red) are detected with consecutive binding of primary *N6*-methyladenosine mAb (CST, D9D9W) and secondary anti-rabbit AlexaFluor 555 Ab. VV proteins are labeled with primary anti-VV antibody (LSBio, LS-C103107) and secondary anti-mouse AlexaFluor 488 Ab. Cell nuclei (blue) are labeled with DAPI. The white scale bar on each image corresponds to 100µm. **(B)** Quantitative analysis of m^6^A-RNA modifications was performed on purified total RNA isolated from MOCK-treated and VV-infected cells utilizing Fluorimetric m^6^A Methylation Kit (ab23349). The relative m^6^A RNA methylation status was calculated as percentage of total input RNA. Shown are the average and sdev from four independent infection experiments.

**
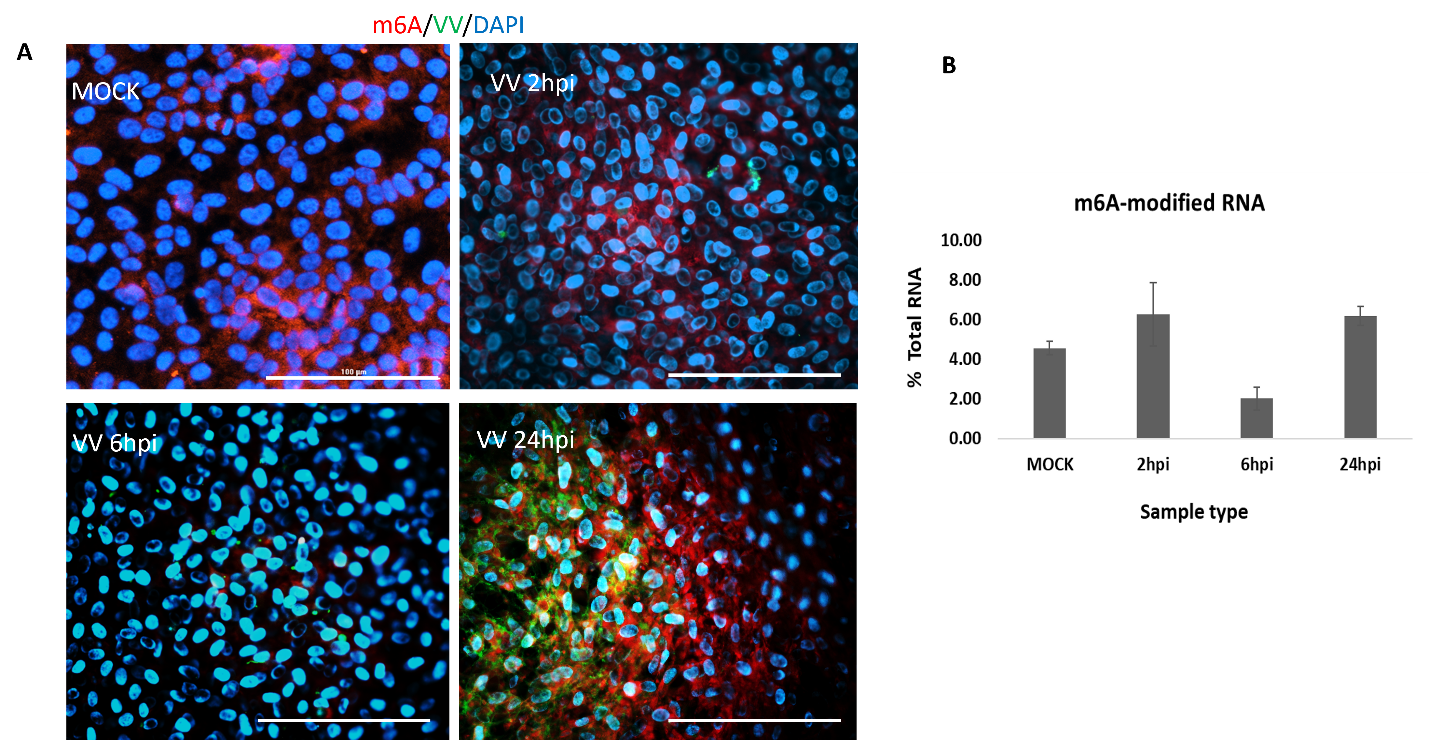
**

**Figure S4. Dispersion plot of host differential gene expressions and host differential m^6^A modifications.** Top panel indicates the overlapping features between the results of RNA-seq and MeRIP-seq analyses. Bottom panel indicates the numbers of loci in each category. Grey colored features indicate non-significant features.


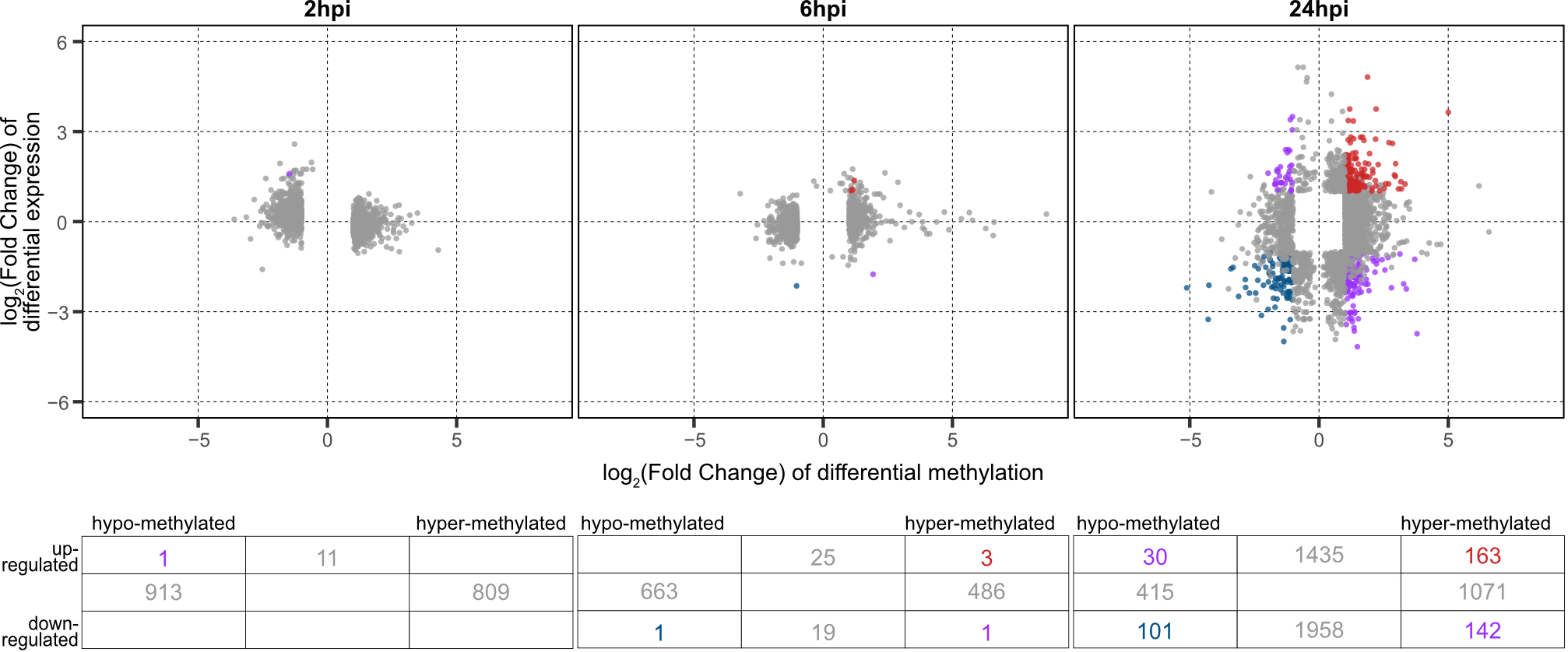


**Figure S5. Changes in m^6^A-modifications detected in *YTHDF1* 5’UTR region.** The pink rectangle indicates the differentially m^6^A-modified region detected in MeRIP-seq analysis. (**A**) Graph of normalized read depth coverage from MeRIP-seq analysis of input and m^6^A-RIP fractions. The top panel illustrates corresponding genomic positions on genic (black) and exonic (grey) regions on the contig NW_023666050.1. The bottom panel illustrates normalized read depth coverages for each biological replicate of either m^6^A-RIP (red) and input (grey) samples across different treatments. (**B**) Graph of the percentage of m^6^A-modified bases from Oxford Nanopore Direct RNA sequencing data. The top panel illustrates corresponding genomic positions on genic (black) and exonic (grey) regions on the contig NW_023666050.1. The bottom panel illustrates the percentage of m^6^A-modified bases over 0% for each genomic location in either m^6^A-RIP (red) and input (grey) samples across different treatments.


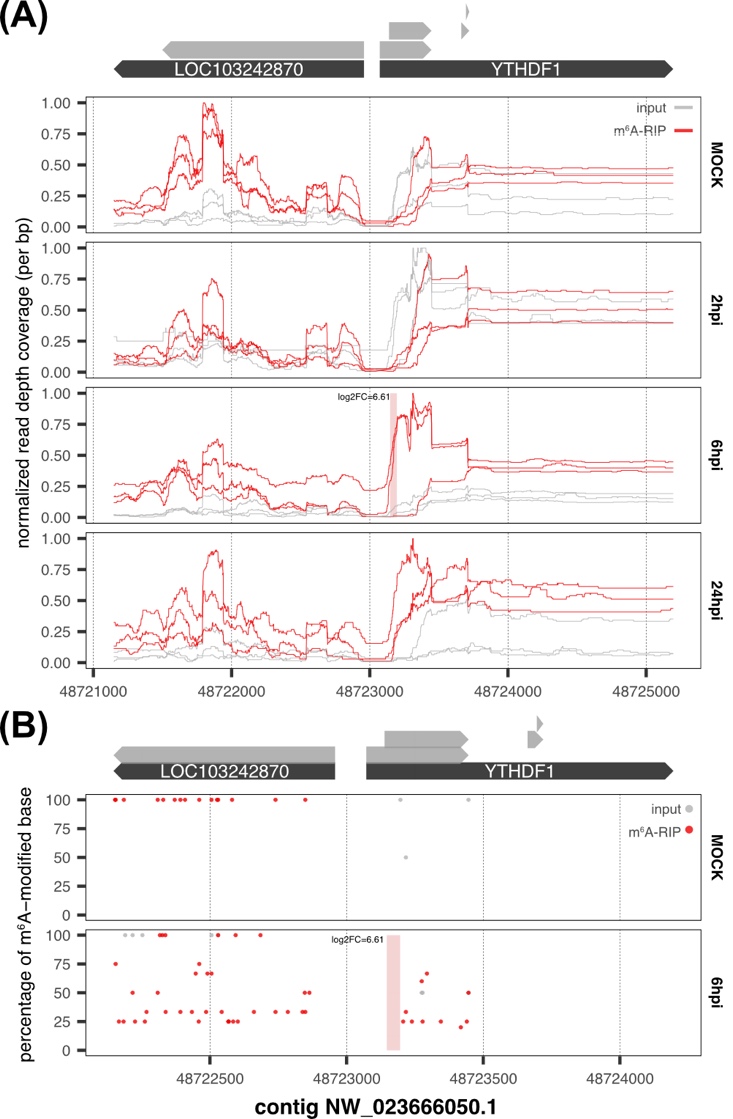


**Figure S6. RT-qPCR analysis for *YTHDF1* transcripts in VV-infected Vero cells.** The *YTHDF1* and *Actin-beta* transcript abundance was calculated in total (input RNA) samples with real-time quantitative PCR. For each sample, *YTHDF1* transcript levels were normalized to *Actin-beta* transcript and fold change was calculated as ratio between VV-infected to MOCK-treated samples. The bar graph presents the average and standard deviations calculated from four independent infection experiments. Red asterisks indicate statistical significance (Wilcoxon rank-sum test *P*-value ≤ 0.05).

**
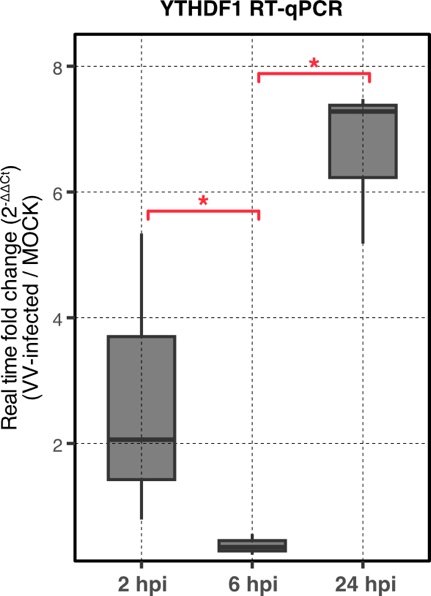
**

**Figure S7. Fluctuations of *YTHDF1* transcripts in VV-infected cells detected by single molecule RNA fluorescence *in situ* hybridization (smRNA-FISH). (A)** Quantitative smRNA-FISH analysis of *YTHDF1* transcripts in Vero cells. Spot counts per cell were calculated from 3,000 image areas of interest that are pooled together from three independent experiments. Each dot indicates *YTHDF1* transcript spot count per cell measured in each image. Color codes MOCK-treated (grey) or VV-infected cells (red). For distinguishable visualization, dots are randomly scattered with ±10% noise. **(B)** Representative fluorescence images of MOCK and VV-infected Vero cells labeled with *YTHDF1* -specific nucleotide probes RNA-FISH detection of *YTHDF1* transcripts (yellow) were performed using 40 RNA probes. Cell nuclei (blue) are labeled with DAPI. The white scale bar on each image corresponds to 10µm.

**
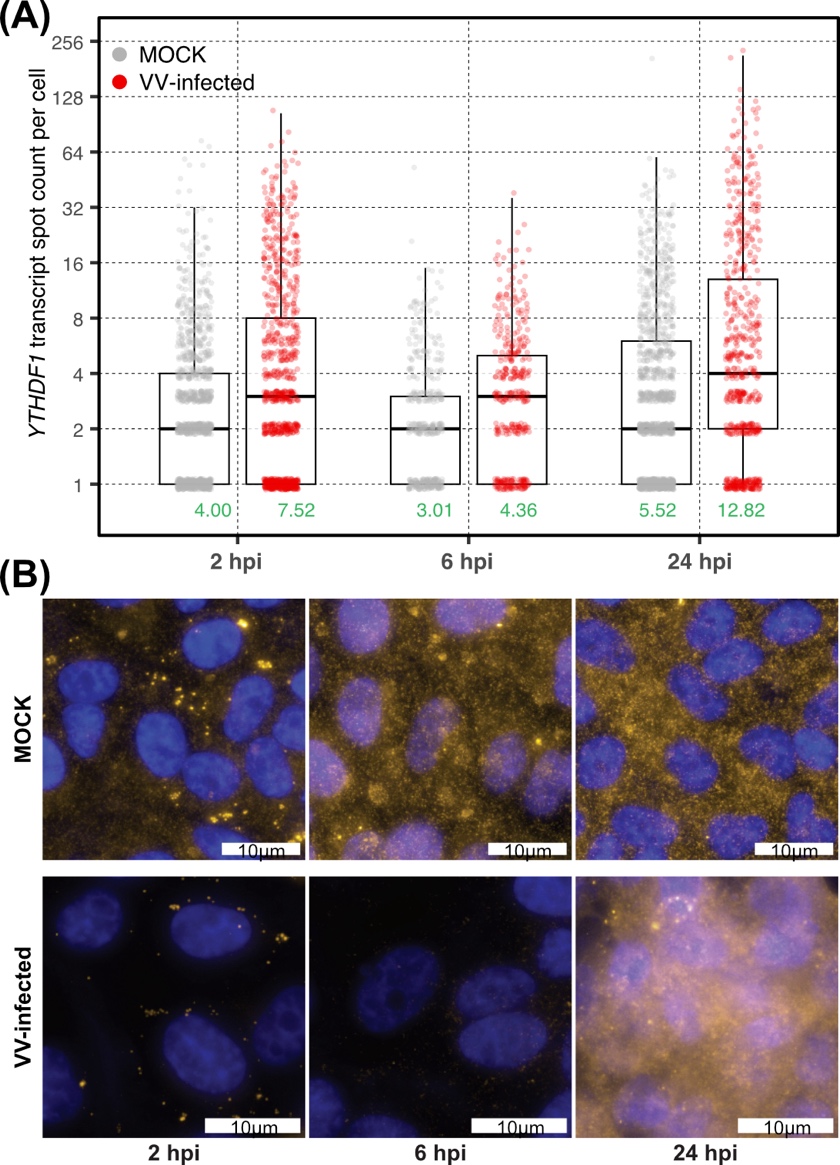
**

**Figure S8. Vaccinia virus spread within monolayers of Vero cells.** Fluorescence immunocytochemistry time-course analysis of VV replication in Vero cells was performed with rabbit polyclonal anti-VV antibody (ab35219) detecting several viral proteins (shown in orange). Cell nuclei are labeled with DAPI (shown in blue). The white scale bar corresponds to 50 μm. Shown are representative of four independent infection experiments images for early (two hours post-infection, 2 hpi), mid (6 hpi) and late (24 hpi) stages of virus replication.

**
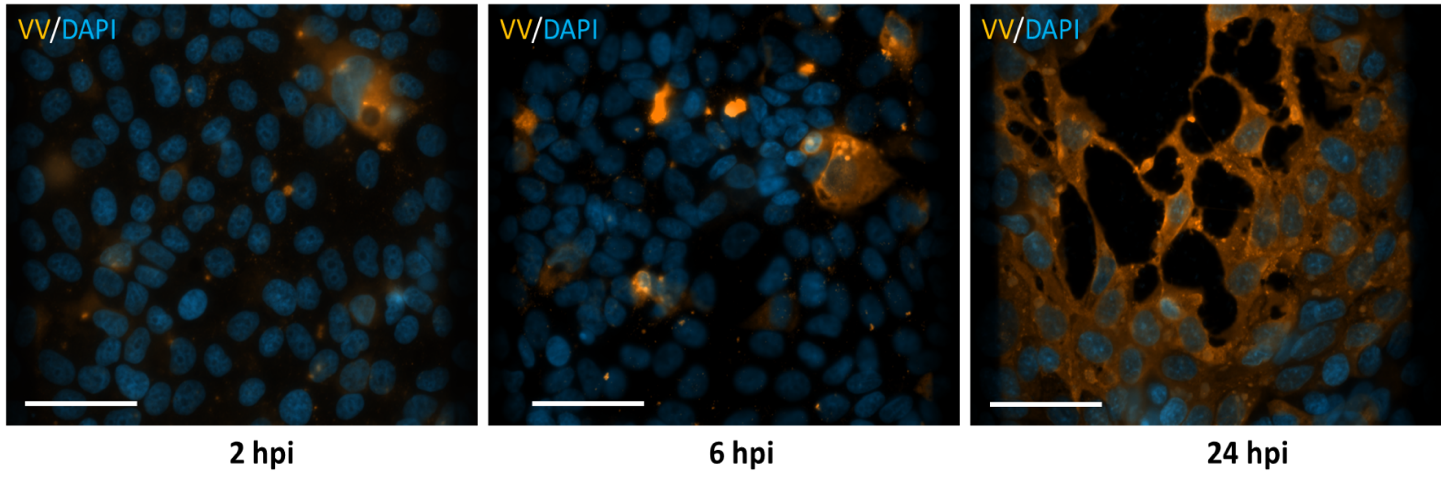
**

**Figure S9. Label-free MS/MS proteomics analysis of VV-infected human skin endothelial cells (HMEC-1) reveal co-expression of YTHDF1 and ARHGEF2.** Average protein abundances determined with Sequest HQ and Percolator using default settings and a precursor mass error tolerance of 15 ppm. Statistics were derived from three independent experimental samples for peptides mapped to Uniprot IDs ARHGEF2 (Q92974) and YTHDF1 (Q9BYJ9). FDR-adjusted *P*-values for ARHGEF2 and YTHDF1 peptide abundance ratios were determined as high confidence ranging from less than 0.01 to 1.07E-16, respectively.

**
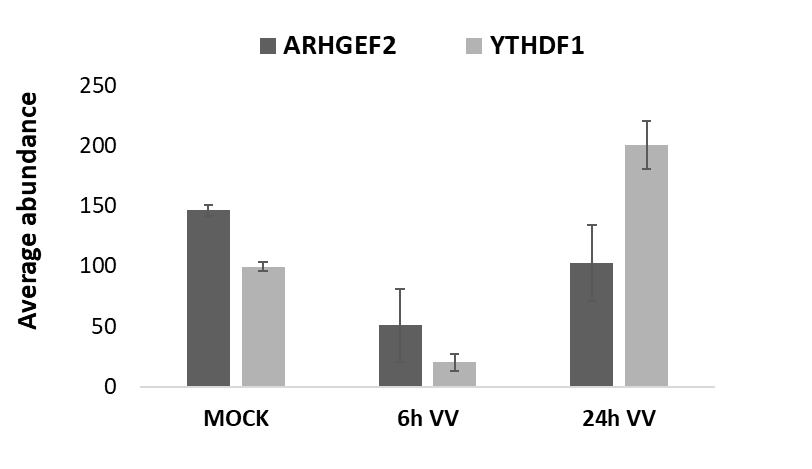
**

**Figure S10. RNAi knockdown of *YTHDF1* restricts Vaccinia virus replication in human skin endothelial cells.** HMEC-1 monolayer cultures, treated with siRNA (20 nM) targeting YTHDF1 transcripts (siRNA-YTHDF1) or non-targeting siRNA duplexes (siRNA-NT), were infected with VV (MOI 1) for 24 hours. Fluorescence immunocytochemistry imaging with rabbit polyclonal anti-VV antibody (ab35219) shows significant reduction in virus replication within HMEC-1 cultures with RNAi-depleted YTHDF1. VV-infected cells are depicted in red with nuclei labeled with DAPI (blue). The white scale bars correspond to 200 μm.

**
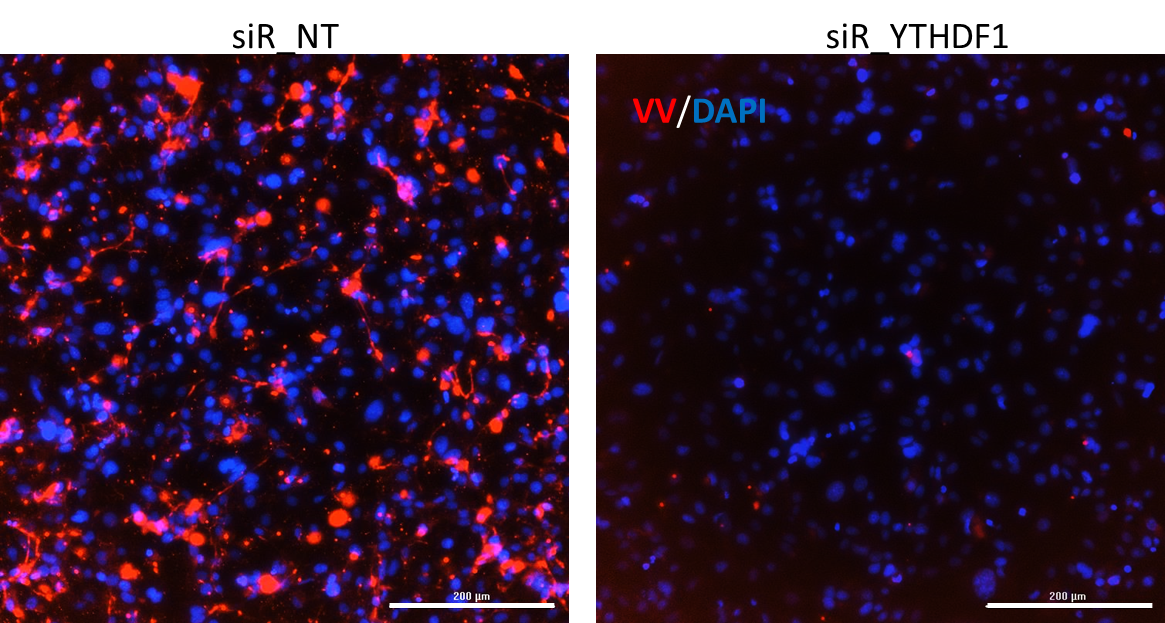
**

**Figure S11. Real-Time quantitative PCR evaluation of FBXO31 transcript knockdown by RNAi.** Successful reduction of *FBXO31* transcripts at 24 hours post-delivery of siRNA targeting *FBXO31* transcripts (siRNA-FBXO31) in HMEC-1 cells was determined with reverse transcription-quantitative PCR using TaqMan probes to human *FBXO31* and *AKT1* transcripts. *AKT1* was used as a negative control for siRNA-*FBXO31* target specificity. Fold changes were measured from three independent experiments, normalized to *beta-Actin,* then calculated relative to control cells treated with non-targeting scramble siRNA (siRNA-NT). When normalized to siRNA-NT controls, expression of *FBXO31* was significantly reduced to 8.96E-02 in siRNA-FBXO31-treated cells, while expression of an off-target gene *AKT1* remained unchanged at 1.22 (Wilcoxon rank-sum test *P*-value=4.00E-02).

**
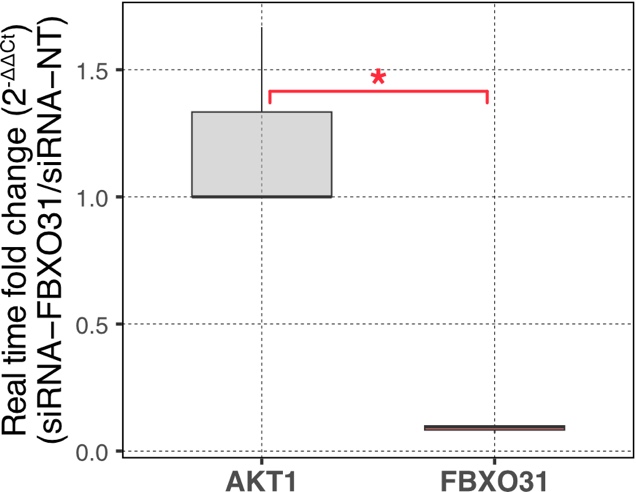
**

**Supplemental References**

1. Ke, S., et al., *A majority of m^6^A residues are in the last exons, allowing the potential for 3′ UTR regulation.* Genes & development, 2015. **29**(19): p. 2037-2053.

2. Rai, S.K., et al., *Candidate tumor suppressor HYAL2 is a glycosylphosphatidylinositol (GPI)-anchored cell-surface receptor for jaagsiekte sheep retrovirus, the envelope protein of which mediates oncogenic transformation.* Proceedings of the National Academy of Sciences, 2001. **98**(8): p. 4443-4448.

3. Zona, L., et al., *HRas signal transduction promotes hepatitis C virus cell entry by triggering assembly of the host tetraspanin receptor complex.* Cell host & microbe, 2013. **13**(3): p. 302-313.

4. Lupberger, J., et al., *EGFR and EphA2 are host factors for hepatitis C virus entry and possible targets for antiviral therapy.* Nature medicine, 2011. **17**(5): p. 589-595.

5. Chen, J., et al., *Ephrin receptor A2 is a functional entry receptor for Epstein–Barr virus.* Nature microbiology, 2018. **3**(2): p. 172-180.

6. Collins, S.E., R.S. Noyce, and K.L. Mossman, *Innate cellular response to virus particle entry requires IRF3 but not virus replication.* Journal of virology, 2004. **78**(4): p. 1706-1717.

7. Watson, S.F., N. Bellora, and S. Macias, *ILF3 contributes to the establishment of the antiviral type I interferon program.* Nucleic acids research, 2020. **48**(1): p. 116-129.

8. Nazitto, R., et al., *ILF3 is a negative transcriptional regulator of innate immune responses and myeloid dendritic cell maturation.* The Journal of Immunology, 2021. **206**(12): p. 2949-2965.

9. Chinnadurai, G., et al., *The Significance of the CtBP—AdE1A Interaction during Viral Infection and Transformation.* GtBP Family Proteins, 2007: p. 44-60.

10. Subramanian, T., L.-j. Zhao, and G. Chinnadurai, *Interaction of CtBP with adenovirus E1A suppresses immortalization of primary epithelial cells and enhances virus replication during productive infection.* Virology, 2013. **443**(2): p. 313-320.

11. Kong, X., et al., *Type I interferon/STAT1 signaling regulates UBE2M-mediated antiviral innate immunity in a negative feedback manner.* Cell Reports, 2023. **42**(1).

12. Sun, M., et al., *Akt plays a critical role in replication of nonsegmented negative-stranded RNA viruses.* Journal of virology, 2008. **82**(1): p. 105-114.

13. Ezell, S.A., et al., *The protein kinase Akt1 regulates the interferon response through phosphorylation of the transcriptional repressor EMSY.* Proceedings of the National Academy of Sciences, 2012. **109**(10): p. E613-E621.

14. Dhawan, T., et al., *TRIM26 facilitates HSV-2 infection by downregulating antiviral responses through the IRF3 pathway.* Viruses, 2021. **13**(1): p. 70.

15. Liang, Y., et al., *TRIM26 is a critical host factor for HCV replication and contributes to host tropism.* Science advances, 2021. **7**(2): p. eabd9732.

16. Munier, S., et al., *Exploration of binary virus–host interactions using an infectious protein complementation assay.* Molecular & Cellular Proteomics, 2013. **12**(10): p. 2845-2855.

17. Dodding, M.P., et al., *A kinesin‐1 binding motif in vaccinia virus that is widespread throughout the human genome.* The EMBO journal, 2011. **30**(22): p. 4523-4538.

18. Augustine, T., et al., *Cyclin F/FBXO1 interacts with HIV-1 viral infectivity factor (Vif) and restricts progeny virion infectivity by ubiquitination and proteasomal degradation of Vif protein through SCFcyclin F E3 ligase machinery.* Journal of Biological Chemistry, 2017. **292**(13): p. 5349-5363.

19. Zhu, L., et al., *Ebola virus VP35 hijacks the PKA-CREB1 pathway for replication and pathogenesis by AKIP1 association.* Nature Communications, 2022. **13**(1): p. 2256.

20. Zhao, X., et al., *Hepatitis B virus DNA polymerase restrains viral replication through the CREB1/HOXA distal transcript antisense RNA homeobox A13 axis.* Hepatology, 2021. **73**(2): p. 503-519.

21. Tomalka, J.A., et al., *The transcription factor CREB1 is a mechanistic driver of immunogenicity and reduced HIV-1 acquisition following ALVAC vaccination.* Nature immunology, 2021. **22**(10): p. 1294-1305.

22. Yang, Q., et al., *Host HDAC4 regulates the antiviral response by inhibiting the phosphorylation of IRF3.* Journal of Molecular Cell Biology, 2019. **11**(2): p. 158-169.

23. Lu, Y., et al., *Histone deacetylase 4 promotes type I interferon signaling, restricts DNA viruses, and is degraded via vaccinia virus protein C6.* Proceedings of the National Academy of Sciences, 2019. **116**(24): p. 11997-12006.
